# Metabolic Blockade-Based Genome Mining of Sea Anemone-Associated *Streptomyces* sp. S1502 Identifies Atypical Angucyclines WS-5995 A–E: Isolation, Identification, Biosynthetic Investigation, and Bioactivities

**DOI:** 10.3390/md22050195

**Published:** 2024-04-25

**Authors:** Yuyang Wang, Le Zhou, Xiaoting Pan, Zhangjun Liao, Nanshan Qi, Mingfei Sun, Hua Zhang, Jianhua Ju, Junying Ma

**Affiliations:** 1 CAS Key Laboratory of Tropical Marine Bio-Resources and Ecology, RNAM Center for Marine Microbiology, Guangdong Key Laboratory of Marine Materia Medica, South China Sea Institute of Oceanology, Chinese Academy of Sciences, Haizhu District, Guangzhou 510301, China; 2 College of Oceanology, University of Chinese Academy of Sciences, Qingdao 266400, China; 3 Key Laboratory of Livestock Disease Prevention of Guangdong Province, Key Laboratory of Avian Influenza and Other Major Poultry Diseases Prevention and Control, Ministry of Agriculture and Rural Affairs, Institute of Animal Health, Guangdong Academy of Agricultural Sciences, Guangzhou 510640, China; 4 Guangdong Provincial Key Laboratory of Medical Molecular Diagnostics, Institute of Laboratory Medicine, Guangdong Medical University, Dongguan 523808, China

**Keywords:** metabolic blockade-based genome mining, *Streptomyces* sp. S1502, WS-5995, biosynthesis, anticoccidial activity

## Abstract

Marine symbiotic and epiphyte microorganisms are sources of bioactive or structurally novel natural products. Metabolic blockade-based genome mining has been proven to be an effective strategy to accelerate the discovery of natural products from both terrestrial and marine microorganisms. Here, the metabolic blockade-based genome mining strategy was applied to the discovery of other metabolites in a sea anemone-associated *Streptomyces* sp. S1502. We constructed a mutant *Streptomyces* sp. S1502/Δ*stp1* that switched to producing the atypical angucyclines WS-5995 A–E, among which WS-5995 E is a new compound. A biosynthetic gene cluster (*wsm*) of the angucyclines was identified through gene knock-out and heterologous expression studies. The biosynthetic pathways of WS-5995 A–E were proposed, the roles of some tailoring and regulatory genes were investigated, and the biological activities of WS-5995 A–E were evaluated. WS-5995 A has significant anti-*Eimeria tenell* activity with an IC_50_ value of 2.21 μM. The production of antibacterial streptopyrroles and anticoccidial WS-5995 A–E may play a protective role in the mutual relationship between *Streptomyces* sp. S1502 and its host.

## 1. Introduction

Actinobacteria associated with plants or animals in marine environment produce various specialized metabolites (SMs) to compete with other microbes and adapt to the habitat [1,2], making them sources that human beings can use for clinical treat and agricultural production. Generally, the genome of an Actinobacteria usually contains at least 20 biosynthetic gene clusters (BGCs) that are responsible for the production of SMs; however, only limited amounts of BGCs are expressed under laboratorial culturing conditions, hindering the potential of further exploiting “talented strains”. To block this limitation, manipulating the genome is a proven approach to activate lowly expressed or unexpressed BGCs by means of promoter refactoring [3], heterologous expression [4], and the overexpression of specific gene(s) such as regulatory or transport genes [5]. Knocking out the core gene(s) related to identify known metabolites in a strain is a simple way of manipulating the genome that can result in an alternative metabolic flow, which might contribute to the identification of different or new SMs. This strategy has been widely applied to many strains including fungi and bacteria [6,7,8]. Since common and shared precursors are utilized to assemble the main skeletons of most natural products, for example, polyketides use acyl-CoA(s) and malonyl-CoA(s), peptides use amino acids and terpenoids use farnesyl diphosphate (FPP) as building blocks, once the production of main chemical compositions is blocked in a strain, precursors can be otherwise used to generate other molecules; thus, novel SMs may be discovered.

**Figure 1:**
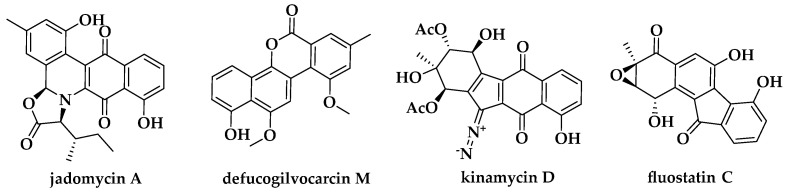
Representative structures of atypical angucyclines.

Atypical angucyclines are polyketides that possess an angular tetracyclic benz[*a*]anthracene scaffold [9], and in some cases have much more complex ring systems that are synthesized by type II polyketide synthase and various post-modification enzymes including a vital JadG-type oxygenase, which directly leads to C–C bond cleavage on ring B/C of angucyclines [10,11]. Extensive studies have been carried out for the discovery and biosynthesis of atypical angucyclines due to their intriguing structures and diversified bioactivities, particularly on jadomycins, gilvocarcins, kinamycins, and fluostatins (Figure 1) [9]. Studies on biosynthetic compounds such as FAD-dependent monooxygenases, oxidoreductases, or other tailoring enzymes in fluostatins biosynthesis [12,13,14] and methyltransferases in gilvocarcins biosynthesis [15] extended the chemical landscape of atypical angucyclines; these enzymes catalyze special reactions and may be applied in synthetic biology to generate useful compounds. Meanwhile, heterologous expression of jadomycin, fluostatin, and kinamycins has been successfully achieved in different *Streptomyces* hosts [16,17,18,19]. On the basis of the knowledge of biosynthetic logic of atypical angucyclines, recently, using a global genome-mining strategy of type II PKS, Zhang’s group reported a type II PKS-containing biosynthetic gene cluster *wsd* which did not match previously reported type II PKS gene clusters in the MIBiG database from a soil-derived strain of *Streptomyces yanglinensis* CGMCC 4.2023, and this led to the discovery of a new compound: WS-5995D [20].

Within our ongoing efforts to discover potential novel secondary metabolites with significant bioactivities or intriguing structures from marine microbial origins using the aforementioned metabolic blockade-based genome mining strategy [7,8], this study focuses on the potential bioactive metabolites of a sea anemone-associated *Streptomyces* sp. S1502. Chemical investigation of *Streptomyces* sp. S1502 led to the identification of three anti-methicillin-resistant *Staphylococcus aureus* (MRSA) halogenated pyrroles (halopyrroles)/streptopyrroles (including a new one, unpublished) [21]. The core gene coding for AMP-dependent ligase for the first biosynthetic step of streptopyrroles was then in-frame deleted to construct the mutant *Streptomyces* sp. S1502/Δ*stp1*. Here, we report (1) five aromatic polyketides belonging to the family of atypical angucyclines, including four known compounds, WS-5995 A–D, and a new one, which herein we term as WS-5995 E, were isolated and identified through 23 L scaled fermentation of the Δ*stp1* mutant; (2) the identification of corresponding BGC *wsm*, proposed biosynthetic pathway and heterologous production in *S. lividans* SBT5 of WS-5995 B–E; (3) probable tailoring and regulatory genes involved in WS-5995 A–E biosynthesis; and (4) the cytotoxic and in vitro anticoccidial bioactivities of WS-5995 A–E.

## 2. Results

### 2.1. Identification of Streptopyrroles from Streptomyces sp. S1502 and the Construction of a Streptopyrroles-Null Mutant

**Figure 2:**
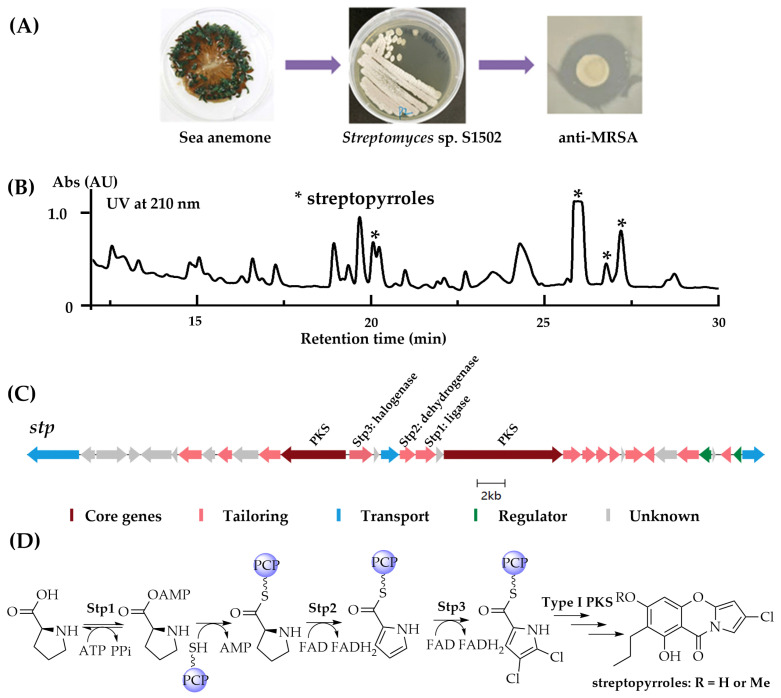
Streptopyrroles from *Streptomyces* sp. S1502 and their biosynthesis. (**A**) Images of the sea anemone host, *Streptomyces* sp. S1502, and the anti-MRSA bioactivity of extract of *Streptomyces* sp. S1502 fermented in RA medium. (**B**) HPLC analysis of extract of *Streptomyces* sp. S1502; peaks marked with * are streptopyrroles. (**C**) The biosynthetic gene cluster *stp* responsible for streptopyrroles biosynthesis; *stp1* for in-frame deletion is located in the middle of the cluster. (**D**) Proposed biosynthetic pathway of streptopyrroles.

In the process of searching for “talented strains” from our in-house symbiotic or epiphytic actinomycetes library associated with marine samples collected from Shenzhen Daya Bay on May, 2021, a strain of *Streptomyces* sp. S1502 isolated from a sea anemone sample caught our eyes during a systematic antibacterial assay screening of nearly 50 Actinobacterial strains, particularly due to its property against methicillin-resistant *Staphylococcus aureus* (MRSA) (Figure 2A) as well as other Gram-positive bacteria. To identify the corresponding compounds responsible for the anti-MRSA activity, three dominant metabolites were isolated from 23 L scaled fermentation of *Streptomyces* sp. S1502 and were identified as streptopyrroles (Figure 2B,D) according to their HR-MS spectrum and 1D NMR spectra (unpublished data) as well as a comparison with published data. This strain was subsequently whole-genome sequenced in order to determine the BGC linked to streptopyrroles biosynthesis and mine its potential abilities to produce other metabolites. The phylogenetic position of strain S1502 was established using autoMLST [22], indicating that it is not related to any other type of strain (Figure S1). The whole genome (~9.3 Mb) of *Streptomyces* sp. S1502 revealed that this strain contains at least 28 putative BGCs for polyketides, non-ribosomal peptides, ribosomal peptides, and terpenoids biosynthesis (Table S4) based on a bioinformatic analysis using antiSMASH 7.0 software [23]. 

Streptopyrroles belong to the family of pyrrole-containing alkaloids, members of this family also contain pyrrolomycin [24], pyoluteorin [25], chlorizidin [26], tetrachlorizine [26], and armeniaspirols [27]. The first shared step of all established biosynthetic pathways related to compounds in this family is the activation and ligation with a carrier protein of proline, which is catalyzed by an amino acid (proline) adenyltransferase such as Arm4 (in armeniaspirol biosynthesis), PrlK (in pyrrolomycin biosynthesis), Tcz16 (in tetrachlorizine biosynthesis), or Clz14 (in chlorizidine biosynthesis). 

**Figure 3:**
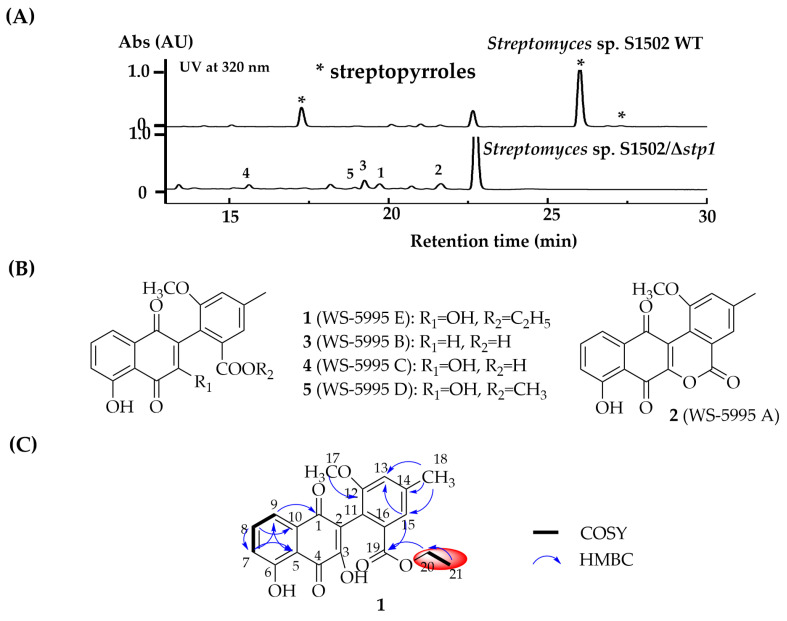
WS-5995 A–E from Δ*stp1* mutant of *Streptomyces* sp. S1502. (**A**) HPLC analysis of extract of *Streptomyces* sp. S1502 and Δ*stp1* mutant. (**B**) Structures of compounds **1**–**5**. (**C**) COSY and HMBC correlations of compound **1**.

Knowing the biosynthetic signature(s) of pyrrole-containing alkaloids, a BGC (BGC11, here designated as *stp*) sharing 37% of its identity with the reported pyrrolomycin BGC (*prl*) containing type I polyketide synthases and other tailoring enzymes were found in the genome of *Streptomyces* sp. S1502 (Figure 2C). Inside the gene cluster of *stp*, *stp1* which shares a strong similarity with *arm4*, *prlK*, *tcz16*, and *clz14* was present along with two genes *stp2* and *stp3* encoding dehydrogenase and halogenase, respectively, catalyzing the following reduction and chlorination of pyrrole, which is coincident with the biosynthesis of the above compounds. Thus, this gene cluster was deduced to be responsible for the biosynthesis of streptopyrrole. To verify this hypothesis, *Streptomyces* sp. S1502/Δ*stp1* (*Streptomyces* sp. S1502S) was constructed through in-frame deletion (Figure S2). A comparison of the metabolic profiling of this mutant with that of in wild-type strain revealed that the production of the streptopyrroles was completely abolished, and significantly increased yields of a series of peaks showing similar UV absorption were observed (Figure 3A). These results confirmed that *stp* is linked to streptopyrroles biosynthesis and a metabolic blockade strategy can be applied to this strain to mine other lowly expressed compounds.

### 2.2. Isolation and Structural Elucidation of Compounds from Streptomyces sp. S1502/Δstp1 Mutant

The mutant *Streptomyces* sp. S1502/Δ*stp1* was fermented at a 23 L scale in RA medium added with HP-20 resins for 7 days. Later, the extracts from the culture broth, resins, and mycelia were combined and subjected to consecutive fractionation using repeated silica gel column chromatography (CC). Prominent peaks in the HPLC analysis were purified by reverse-phase HPLC to produce compounds **1**–**5**. 

Compound **1** was obtained as a yellow solid. High-resolution electrospray ionization mass analysis afforded a [M + H]^+^ ion at *m*/*z* 383.1122 (cald for 383.1125, Figure S3), suggesting that the molecular formula of **1** can be deduced to be that of C_21_H_18_O_7_ and 13 degrees of unsaturation are required. The ^1^H and ^13^C NMR data (Table 1) showed a signal distribution typical for a tetracyclic benz[*a*]anthracene framework. The ^1^H NMR spectrum (Figure S4) displayed signals for five aromatic (*δ*_H_ 7.69, 7.64, 7.53, 7.27, and 6.99) and two methyl (*δ*_H_ 3.77 and 2.44) signals. The combined analysis of ^13^C NMR (Figure S5) and HMBC spectra (Figure S6) suggested the presence of 21 carbons including three carbonyls (*δ*_C_ 185.0, 182.9, and 166.5), 12 aromatic (*δ*_C_ 166.5, 161.4, 157.3, 151.5, 140.4, 137.7, 133.2, 133.2, 131.4, 123.5, 123.2, 122.4, 120.1, 117.9, 116.1, and 113.6), one methylene (*δ*_C_ 61.1), and three methyl (*δ*_C_ 56.3, 21.9, and 14.2) carbons. A 1,2,3-trisubstituted aromatic ring was indicated by diagnostic scalar coupling constants and the observation of COSY correlations (Figure S7) between doublets H-7 (*δ*_H_ 7.23, *J* = 8.4 Hz) and H-9 (*δ*_H_ 7.69, *J* = 7.9 Hz) and the H-8 triplet (*δ*_H_ 7.64, *J* = 8.4 and 7.9 Hz). The HSQC correction (Figure S8) of C-20/H-20 (*δ*_C/H_ 61.1/4.17) and C-21/H-21 (*δ*_C/H_ 14.2/1.19) and the COSY correlation between H-20 and H-21 suggested the presence of an ethyl group, which was connected to the same ester group as WS-5995D.

Compounds **2**–**5** were determined to be the known compounds WS-5995A, WS-5995B, WS-5995C, and WS-5995D, respectively, by analysis of their ^1^H and ^13^C NMR and HR-ESI-MS data (Figures S9–S20) [20,28,29].

### 2.3. Characterization and Heterologous Expression of the wsm Biosynthetic Gene Cluster and Preliminary Investigation into the Genes Involved in Biosynthetic Pathways of Compounds ***1***–***5***

**Figure 4:**
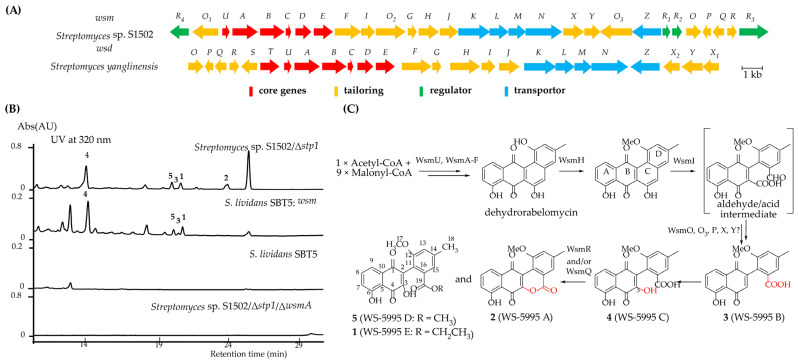
Proposed biosynthesis of WS-5995 A–E. (**A**) Comparison of *wsm* and *wsd* gene clusters and their gene organization. (**B**) Confirmation of *wsm* gene cluster by HPLC analysis. (**C**) Proposed biosynthetic pathway of WS-5995 A–E.

Genome sequence analysis of *Streptomyces* sp. S1502 revealed a contiguous region spanning ~30 kb containing 29 open reading frames (ORFs) that displayed a relatively high similarity and similar organization to the previously reported BGC (Figure 4A, *wsd*; MIBiG ID2712) [20]. Remarkably, a homolog for almost every gene in the *wsd* gene cluster can be found in the *wsm* gene cluster in *Streptomyces* sp. S1502 strain, and every homologue in the WS-5595 BGC (*wsd*) shares a 58–90% amino acid sequence similarity and 46–81% of its identity with its corresponding gene associated with compounds **1**–**5** biosynthesis (Table S5). We noticed that some *Streptomyces* from different origins in public database also harbor this gene cluster with exactly the same gene composition or a highly similar organization (Figure S21), which indicates they are latent producers of the WS-5995 series of compounds.

To verify whether the *wsm* gene cluster is responsible for WS-5995 A–E production, the Δ*wsmA* in-frame deletion mutant was constructed based on the Δ*stp1* mutant (Figure S22). HPLC analysis of the double mutant reveals the abolishment of WS-5995 A–E, proving that *wsm* is related to WS-5995 A–E production (Figure 4B). Some biosynthetic gene clusters of atypical angucyclines have been successfully heterologously expressed in different model microorganisms, thus extending the chemical diversity of this family of compounds and enabling the confirmation of the biosynthetic gene clusters. For example, an environmental DNA fragment containing type II PKS was heterologously expressed in *Streptomyces albus* J1074, resulting in the isolation of fluostatins [16]. Coincidentally, *fls* from the marine-derived fluostatins-producing strain *Micromonospora rosaria* SCSIO N160 was introduced into *Streptomyces coelicolor* YF11, leading to the production of a new dimer fluostatin, difluostatin A [18]. Also, the gene cluster *alp* identified from *S. galtieri* Sgt26 was transferred into the heterologous host *S. albus* J1074 and the recombinant strain produced kinamycins [19]. 

To further link the relationship between WS-5995 A–E production and the proposed *wsm* gene cluster, heterologous expression of the *wsm* cluster in *Streptomyces lividans* SBT5 [30] and *Streptomyces atratus* ZH16NSEP (our genetic-engineered marine-derived chassis cell for a scaled heterologous expression platform of marine microbial bioactive metabolites) [31] was performed. A bacterial artificial cosmid library of the S1502 strain was constructed. We screened a recombinant plasmid pBAC/1-9A carrying the intact *wsm* BGC from the bacterial artificial chromosome (BAC) library of *Streptomyces* sp. S1502/Δ*stp1*. The plasmid pBAC/1-9A was then introduced into the two heterologous hosts, respectively, via triparental intergeneric conjugation, generating *Streptomyces lividans* SBT5: pBAC/1-9A and *Streptomyces atratus* ZH16NSEP: pBAC/1-9A. HPLC analysis of the heterologous strain fermentation extract revealed that the *wsm* cluster could be solely successfully expressed in *S. lividans* SBT5 and could stably produce WS-5995 B–E, and while WS-5995 A was not detected, the yields of WS-5995 B–E were relatively lower compared to the wild-type producer (Figure 4B).

According to bioinformatic analysis of each gene in *wsm* BGC, the biosynthesis of WS-5995 A–E is proposed (Figure 4C). A common biosynthetic pathway of aldehyde/acid intermediate of atypical angucyclines is proposed as follows: WsmA, WsmB, and WsmC utilize one acetyl-CoA unit and nine malonyl-CoA units to form the initial 20-carbon polyketide chain, which is reduced at position C-9 by WsmD and cyclized by WsmE and WsmU, before further functioned by WsmF and WsmT; then, the common intermediate dehydrorabelomycin is formed. Subsequently, the methyltransferase WsmH is proposed to hydroxylate the -OH group of ring D prior to the GilOII/JadG homolog WsmI (Figure S23) catalyzing the C-C bond cleavage of ring C to form the aldehyde/acid intermediate, as once the carboxyl group is formed, it might react with the hydroxylation group to form an ester product, similar to gilvocarcins biosynthesis [11], while such products were not isolated from *Streptomyces* sp. S1502/Δ*stp1* mutant. Decarboxylation and oxidation of the aldehyde group of the aldehyde/acid intermediate leads to the formation of WS-5995 B, and hydroxylation on the position of decarboxylation forms WS-5995 C, which are probably catalyzed by WsmO/P/Q/R; the four protein homologs are also present in *wsd* gene cluster. Last, intramolecular esterification of WS-5995 C and intermolecular esterification of WS-5995 C and methanol/ethanol were carried out to form WS-5995 A and WS-5995 D/E.

The results of the deletion of the KS-coding gene *wsmA* and heterologous expression of *wsm* gene cluster unambiguously confirmed the correlation between WS-5995 A–E production and the *wsm* gene cluster; we next sought to identify putative genes involved in the final steps of WS-5995 A–E biosynthesis. A question can be raised about whether WS-5995E (**1**) is an artificial product, as during the extraction process, the reason that the ethanol was used, pure WS-5995 C (**4**) was dissolved in methanol and ethanol for 72 h, respectively, we did not detect the production of WS-5995A (**2**), D (**5**), or E (**1**) (Figure S24). To further verify this, *Streptomyces* sp. S1502 was re-fermented in RA medium without the addition of resins, and it was extracted with butanone. The HPLC analysis results showed that WS-5995 A–E were produced too. These two observations indicate that WS-5995A, D, and E are not spontaneous products, indicating that the formation of an ester bond is more likely to be an enzymatic process. 

**Figure 5:**
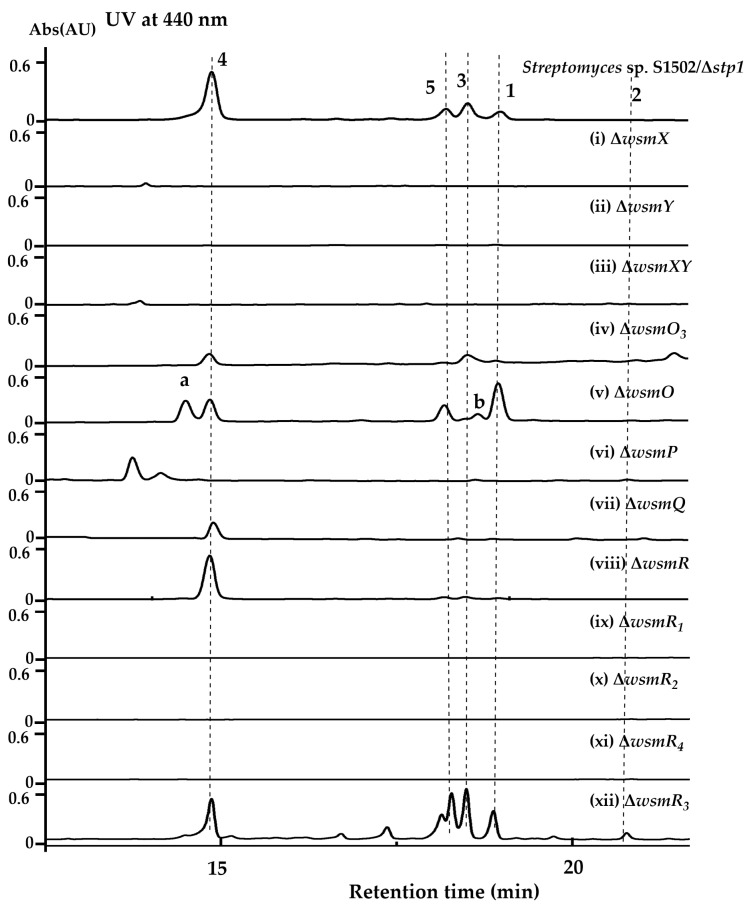
HPLC analysis of mutants derived from *Streptomyces* sp. S1502/Δ*stp1.* **1**–**5** stand for compounds mentioned in the manuscript.

To identify the function of tailoring and regulatory genes involved in WS-5995 A–E biosynthesis, the *wsm* gene cluster and *wsd* gene cluster were re-examined and compared using a clinker [32] (Figure S25). Some genes are relatively special in *wsm*/*wsd* gene clusters compared to other atypical angucycline biosynthetic gene clusters, including *wsmO*, *P*, *Q*, *R*, *X*, *Y*, and *O_3_* (in *wsm*). These genes might be involved in the tailoring steps in the creation of WS-5995 A–E, and therefore, were individually deleted (Figure S26). The corresponding mutants were fermented; the Δ*wsmO* and Δ*wsmO_3_* mutants retained the production of WS-5995 A–E, the former also produced two new products (a and b), while the latter had a lower yields of the five compounds; the Δ*wsmP*, Δ*wsmX* and Δ*wsmY* mutants abolished the production of WS-5995 A–E, and the Δ*wsmR* and Δ*wsmQ* mutants only produced WS-5995 C (**4**) (Figure 5i–viii). According to the HPLC analysis of the above mutants, all seven of the genes have been preliminarily deduced to be involved in WS-5995 biosynthesis; *wsmO_3_* may have a homolog in the *Streptomyces* sp. S1502 genome that has a complemental function to WsmO_3_; WsmP, WsmX, and WsmY are involved the tailoring steps of the aldehyde/acid intermediate; WsmR and WsmQ may participate in the transformation of WS-5995 C (**4**) to WS-5995 A (**2**); and WsmO might catalyze an unknown reaction. Combining these results with the result that the heterologous expression of *wsm* gene cluster did not produce WS-5995 A (**2**), indicated that the enzyme catalyzing WS-5995 C (**4**) to WS-5995 A (**2**) might be located outside of the gene cluster.

The role of the four regulatory genes *wsmR_1_*-*R_4_* in WS-5995 A–E biosynthesis was also characterized to provide a mutant that might have higher yields of WS-5995 A–E. The four genes were individually deleted (Figure S26) and the mutants were fermented. As seen from the HPLC analysis, *wsmR_1_*, *R_2_*, and *R_4_* have been preliminarily deduced to have a positive role, while it appears that *wsmR_3_* has a negative role in WS-5995 A–E biosynthesis (Figure 5ix–xii). Thus, the Δ*wsmR_3_* mutant is a relatively high-producing strain of WS-5995 A–E.

### 2.4. Bioactivities of WS-5995 A–E

Four human cancer cell lines (MCF-7, BT-549, RBE and A549) and two normal human cell lines (NCM-46 and Huvec) were used to test the cytotoxicity of compounds **1**–**5** (Table 2). Compounds **1**, **4,** and **5** did not exhibit cytotoxic effects against the abovementioned cell lines, while both compounds **2** and **3** showed slight cytotoxicity against the RBE, NCM-460, and Huvec cell lines, with IC_50_ values of 21.39, 40.84, and 31.24 μM for compound **2** and 16.79, 18.47, and 7.67 μM for compound **3**, respectively. In addition, compound **3** was also found to show cytotoxicity against MCF-7 and BT-549 cell lines, with IC_50_ values of 19.60 and 5.97 μM, respectively. From the structure and the activities, we proposed that the ester bond might be important to cytotoxic activity, and hydroxylation at C-3 can lead to the removal of cytotoxic activity.

A previous study established that WS-5995 A (**2**) and B (**3**) showed excellent protective activity against *Eimeria tenella* infections in animal experiments [29]. However, the in vitro anticoccidial activity is still lacking experimental data. Compounds **1**–**5** were evaluated for their in vitro anti-*E. tenella* bioactivity. WS-5995 A (**2**) had clear anti-*E. tenella* activity with an IC_50_ value of 2.21 μM, and while compound **4** is not active, compounds **1** and **5** only exhibited activity at high concentrations. These results coincide with the animal experiments reported previously. The in vitro anticoccidial activity showed that the lactone bond of WS-5995 A is vital to this bioactivity.

## 3. Materials and Methods

### 3.1. General Experimental Procedures

DNA isolation and manipulation were carried out following standard procedures for *E. coli* and *Streptomyces*. Reagents for polymerase chain reactions (PCRs) were purchased from Takara Co. (Dalian, China) and Trans Gene Co. (Beijing, China). The plasmid extraction kit and gel extraction kit were purchased from Omega (Beijing, China). The media used for fermentation were purchased from Guangdong Huankai Microbial Technology Co., Ltd. (Guangzhou, China). Luria–Bertani (LB) broth used for *E. coli* cultivation was purchased from Oxoid Co., Ltd. (Hants, UK). Organic solvents for compounds isolation and purification were bought from Guangzhou Chemical Regent Factory (Guangzhou, China) or J&K Scientific Co., Ltd. (Beijing, China). All chemicals and solvents were of analytical or chromatographic grade. All primers and reagents used in this work were purchased from Sangon Bio–Pharm Technology Co., Ltd. (Shanghai, China).

Analytical HPLC was performed using an ODS (2) column (150 mm × 4.6 mm, 5 μm) on an Agilent 1260 HPLC system. Semi-preparative HPLC was performed on an Agilent 1260 HPLC system equipped with diode array detector (Agilent Corporation, Santa Clara, CA, USA), using ODS-A column (10 mm × 250 mm, 5 μm, YMC, Co., Ltd., Kyoto, Japan). High-resolution mass spectrometry data were measured with a MaXis Q-TOF mass spectrometer (Bruker, Billerica, MA, USA). NMR spectra were recorded on a Bruker AVANCE 500 or a Bruker AVANCE 700 spectrometer (Bruker, Billerica, MA, USA). UV spectra were recorded with a UV-2600 spectrometer (Shimadzu, Japan). IR spectra were measured with PerkinElmer FT-IR 1760X. Optical rotations were measured using a Polartronic 341 HNQWS Fully Automatic High Resolution Polarimeter.

### 3.2. Strains, Plasmids, and Culture Conditions

The strain S1502 was isolated from a sea anemone sample collected from Shenzhen Daya Bay in May 2021, and it was identified as *Streptomyces* sp. based on its morphological characteristics as well as phylogenetic analysis of the 16S rRNA gene sequence.

*Streptomyces* sp. S1502 used for genetic engineering was cultivated on MS medium (20 g/L soybean meal, 20.0 g/L d-mannitol, and 20.0 g/L aga, pH 7.2–7.4) for sporulation. *Streptomyces* sp. S1502 and its mutants were cultivated in RA medium (malt extract 10 g/L, maizena 5 g/L, soluble starch 20 g/L, maltose 10 g/L, glucose 10 g/L, trace elements 100 μL/L, sea salt 30 g/L, CaCO_3_: 2 g/L, pH 7.2~7.4) for secondary metabolites production. *E. coli* Bw25113/pIJ790 was used for PCR-targeting, and *E. coli* ET12567/pUZ8002 was used for conjugation. LB medium was used for *E. coli* and appropriate antibiotics were added at a working concentration of 50 μg/mL apramycin (Apr), 50 μg/mL kanamycin (Kan), 50 μg/mL trimethoprim (TMP) and 25 μg/mL chloroamphenicol (Cm/Chl) when necessary. Bacterial strains and plasmids used and constructed in this study are listed in Table S1.

### 3.3. Genome Sequencing and Bioinformatic Analysis

The secondary metabolite BGCs of *Streptomyces* sp. S1502 were identified and analyzed using the online software antiSMASH version 7.1.0 [23]. The *orf* assignments and their proposed functions were analyzed using the online FramePlot 4.0beta (http://nocardia.nih.go.jp/fp4/, accessed on 7 June 2022). Functional prediction and domain annotation of *orfs* were accomplished using BLAST software (http://blast.ncbi.nlm.nih.gov/, accessed on 7 June 2022). Sequence alignments were achieved using MEGA 7.0 and Clustal X.

### 3.4. Construction of the Δstp1 and Mutants Based on the Δstp1

λ-RED mediated PCR-targeting method was applied in this study to inactivate the targeted genes [31,33]. First, a 1384 bp apramycin resistance gene as a PCR template was obtained through digestion of plasmid pIJ773 by *Hin*dIII and *Eco*RI. Then, each of the apramycin resistance gene cassettes (*aac(3)IV-oriT*) was amplified by PCR using the obtained template with designed primers listed in Table S2. Subsequently, the PCR-targeting and conjugation processes were carried out as described previously [31]. The construction process of in-frame deletion *strp1* was similar to our previous report and can simply be described as follows. 

For *stp1* in-frame deletion, the Supercos I vector-based cosmid 10-11F harboring the partial streptopyrrole cluster containing *stp1* was used. The apramycin resistance gene cassette (*aac(3)IV-oriT*) was amplified by PCR using the obtained template (del-*stp1*-F and del-*2970*-R) with the designed primers listed in Table S2. The apramycin resistance cassette was introduced into *E. coli* BW25113/pIJ790/10-11F by chemical transformation to disrupt the homologous gene region to generate cosmid 10-11F-a. The mutated cosmid 10-11F-a was digested with *Spe*I and subsequently self-ligated to generate 10-11F-b which was verified with the primers listed in Table S2. Then, the kanamycin resistance gene of the cosmid 10-11F-b was replaced by the *aac(3)IV-oriT* cassette and transferred to non-methylating *E. coli* ET12567/pUZ8002, and then introduced into *S.* sp. S1502 by conjugation. Exconjugants were verified with the primers listed in Table S2 to generate single crossover mutants and then to generate double-crossover mutant indel-Δ*stp1* after two generations of relaxed cultivation. 

The construction of the Δ*wsmA* mutant followed the same process of Δ*stp1* mutant based on the Δ*stp1* mutant. Other mutants were constructed by replacing the deleted fragments of cosmids 13-4D or 13-5D with the apramycin resistance gene cassette (*aac(3)IV-oriT*).

### 3.5. Heterologous Expression of the wsm Gene Cluster in S. lividans SBT5

The bacterial artificial chromosome (BAC) genomic library of *Streptomyces* sp. S1502 was constructed by Eight Star Biotech [34] (http://www.eightstarsbio.com, accessed on 1 January 2023) using the vector pMSBBAC2 and stored in *E. coli* DH10B. The BAC library was screened by PCR using the primers to afford the desired colonies pBAC1-9C/1-16A, which contain the intact *wsm* BGC. pBAC1-9C/1-16A was used for conjugation with *S. lividans* SBT5 and *S. atratus* SCSIO ZH16NSEP using the triparental conjugation methods and cultured on MS or ISP4 medium. The correctness of the conjugants was verified by PCR analysis.

### 3.6. Small-Scale Fermentations of Streptomyces sp. S1502 and Its Mutants

*Streptomyces* sp. S1502 and its mutants were inoculated on MS medium supplemented with 2–3% agar and grown for 7 days. The mycelium and spores (1 cm^2^) of the WT and mutant strains were inoculated into a 250 mL flask with 50 mL of RA medium with 3% XAD-16 at 28 °C and 200 rpm for 7 days. The cultures were each harvested and extracted with 100 mL of butanone, evaporated to dryness, and dissolved in MeOH for HPLC-UV-DAD analysis using a reversed phase column SB-C18, 5 μm, 4.6 × 150 mm (Agilent) with UV detection at 210, 280, 320, and 440 nm under the following program: solvent system (solvent A, 15% acetonitrile in water supplemented with 0.1% acetic acid; solvent B, 85% acetonitrile in water supplemented with 0.1% acetic acid); 20% B to 80% B (linear gradient, 0–20 min), 80% B to 100% B (linear gradient, 20–21.5 min), 100% B (21.5–27.0 min), 100% B to 0% B (27.0–27.1 min), 0% B (27.1–30.0 min); flow rate was set at 1 mL min^−1^. 

### 3.7. Large-Scale Fermentations of Streptomyces sp. S1502 and Δstp1 Mutant

A portion of mycelium and spores of the wild-type strain or the Δ*stp1* mutant of *Streptomyces* sp. S1502 were seeded into 50 mL RA medium in a 250 mL flask and a 23 L scaled fermentation was carried out. After incubation in a shaker at 28 °C, 200 rpm for 48 h, 25 mL of the culture was transferred into a 1 L flask containing 200 mL RA medium and then cultured at 28 °C and 200 rpm for 7–8 d. The growth culture was centrifuged at 3900 g for 10 min to yield supernatant and mycelium cake. The supernatant, mycelium, and resins were independently extracted by twice the volume of butanone, acetone, and ethanol three times, respectively. The organic extracts were combined and concentrated to dryness, yielding a syrupy residue.

The residue was subjected to a normal phase silica gel (100–200 mesh) column eluted with CHCl_3_–MeOH (100:0, 99:1, 98:2, 97:3, 95:5, 93:7, 90:10, 80:20, 70:30, 50:50, 0:100, *V*/*V*) to give eleven fractions, A1–A11, respectively. Fr. A1 was loaded onto a Sephadex LH-20 column, and eluted using an isocratic mix of CHCl_3_−MeOH mixture of 5:5 to get 20 factions B1–B20; Fr. B1 was separated by semi-preparative HPLC eluted with MeCN/H_2_O at a flow rate of 2.5 mL/min to yield compound **5** (WS-5995 D, 6 mg, yield: 0.26 mg/L); Fr. B3–B8 were combined and recrystallized to give compound **2** (WS-5995 A, 12 mg, yield: 0.5 mg/L); Fr. A2 was subjected a normal phase silica gel (100–200 mesh) column eluted with CHCl_3_–MeOH (100:0, 97:3, 95:5, 93:7, 90:10, 80:20, 70:30, 50:50, 0:100, *V*/*V*) to give 9 fractions, C1–C9; Fr. C1 and C2 were combined and separated by semi-preparative HPLC eluted with MeCN/H_2_O at a flow rate of 2.5 mL/min to yield compounds **3** (WS-5995 B, 7 mg, yield: 0.30 mg/L) and **1** (WS-5995 E, 4 mg, yield: 0.17 mg/L). Fr A4 was subjected a normal phase silica gel (100–200 mesh) column eluted with CHCl_3_–MeOH to give eleven fractions D1–D7, respectively, and Fr. E3 and E4 were combined and separated by semi-preparative HPLC eluted with MeCN/H_2_O at a flow rate of 2.5 mL/min to yield compound **4** (WS-5995 C, 12 mg, yield: 0.46 mg/L).

### 3.8. Cytotoxic Activities of Compounds ***1***–***5***

Compounds **1–5** were tested for their cytotoxic activities against different normal and tumor human cell lines, the MTT assay was used to assess cytotoxicity as described previously. Briefly, different tumor cells and normal cells were seeded onto 96-well plates at the appropriate density and allowed to attach overnight. Drugs at various concentrations were added to the culture medium and cells were cultured for another 68 h at 37 °C. MTT (5 mg/mL, 20 μL/well) was then added to the wells for additional 4 h, and the medium was discarded before 150 μL of DMSO was added to dissolve the MTT-formazan crystals. Absorbance was measured at 540 nm, with background subtraction at 670 nm using Model 550 Microplate Reader (Bio-Rad, Hercules, CA, USA). The Bliss method was used to calculate the half maximal (50%) inhibitory concentration (IC_50_) values. Data represent the mean ± SD of at least three independent experiments.

### 3.9. In Vitro Anticoccidial Activity

The number of *Eimeria tenella* spores and the cells were seeded in a 24-well plate at a ratio of 1:1 and incubated for 6 h, and the medium was discarded and washed with PBS three times. Then, 500 μL of medium was added to each well, followed by the addition of the medium containing the compounds, the blank control group (no spores and no compounds) and positive control group (with spores but no compound) were set, and 10 μM of sulfaclozinium chloride sodium served as a control drug, and three replicates were set up. The culture remained in an incubator containing 5% CO_2_ at 38 °C for 48 h. After 48 h, the medium was discarded and washed with PBS three times, and the total RNA of each well was extracted and reverse-transcribed into cDNA. qPCR was used to detect the effects of the compounds on the growth and development of *E. tenella*.

## 4. Discussion and Conclusions

In the long history of evolution, actinomycetes and their related marine or terrestrial animals might have had a mutual relationship, for the reason that they produce antibiotics to protect the hosts and utilize the excellent environment provided by the host, as can be seen from some other studies [2,35,36]. To take advantage of this, natural products from these microorganisms have drawn much more attention as they seem to be more potent and can be developed into drugs. The marine symbiotic microorganisms harbor vast BGCs to help their host adapt to the extreme marine environment and have great potential to produce various novel natural products. However, only limited BGCs are active in one strain and many natural products are thus only rediscovered under conventional experimental cultivation conditions. With the rapid advancement of genome sequencing and the development of bioinformatics, numerous genome mining strategies have been applied to discover novel natural products, but many attempts have been in vain. It is worth mentioning that one of the genome mining strategies, metabolic blockade-based genome mining, is efficient and can be used to discover novel natural products [1,6,7,8].

Atypical angucyclines are a type of natural product possessing an angular tetracyclic benz[*a*]anthracene scaffold [9] synthesized by type II polyketide synthase and various post-modification enzymes. The WS-5995 type of compounds belonging to atypical angucyclines were isolated from the *Streptomyces* sp. S1502 by metabolic blockade-based genome mining strategy in this study, and our further study revealed that many post-modification enzymes’ genes are involved in the biosynthesis of WS-5995. The characterization of regulatory genes in *wsm* BGC led to the generation of a high titer production of the engineered WS-5995 strain, which will be helpful for the further study of WS-5995 compounds. In addition, the bioactivity test of these five compounds showed that WS-5995 A has significant anticoccidial activity and is non-toxic against the tested cell lines, which indicated that it has great potential to be developed into anticoccidial drugs. In addition, WS-5995 compounds exhibited broad-spectrum antibacterial and anticoccidial activities, respectively; in addition, WS-5995B was also reported to have antifungal activity [28], which led us to speculate here that this bacterium may use these compounds to protect the sea anemone host from infections from specific pathogenic bacteria/fungi and protozoan, explaining the plausible ecological functions of these compounds produced by this sea anemone-associated bacterium. 

In summary, five atypical angucyclines, WS-5995 A–E, were discovered through the metabolic blockade-based genome mining strategy from a sea anemone-associated *Streptomyces* sp. S1502 Δ*stp1* mutant, among which WS-5995 E is a new compound. The BGCs (*wsm*) of WS-5995 A–E were identified by combining gene-knockout experiments and heterologous expression studies, and some tailoring and regulatory genes involved in WS-5995 A–E biosynthesis were also investigated. The gene knockout results indicated that WsmO_3_, O-R, X, and Y are involved in WS-5995 A–E biosynthesis, but their specific roles are yet to be confirmed; the regulatory gene *wsmR3* had a negative effect on the production of WS-5995 A–E, and the deletion of this gene clearly increased the yield of WS-5995 A–E. The gene cassette *wsmO*-*R* can be regarded as a unique signature compared to other atypical angucyclines BGCs.

## Figures and Tables

**Table 1:** ^13^C (125 MHz) and ^1^H (500 MHz) NMR data for compound **1** (CDCl_3_).

Position	Compound		
	WS-5995 E (1)		
	*δ*_C_ Type	*δ*_H_ Mult. (*J* in Hz)	HMBC
1	182.9, C	-	-
2	117.9, C	-	-
3	131.4, C	-	-
3-OH	-	-	-
4	185.0, C	-	-
5	113.6, C	-	-
6	161.4, C	-	-
6-OH	-	11.21, s	113.6 (5); 123.2 (7); 137.7 (8); 161.4 (6)
7	123.2, CH	7.23 (d, *J* = 8.4 Hz)	113.6 (5); 120.1 (9)
8	137.7, CH	7.64 (dd, *J* = 8.4, 7.9 Hz)	133.2 (7); 161.4 (10)
9	120.1, CH	7.69 (d, *J* = 7.9 Hz)	113.6 (5); 123.2 (7); 182.9 (1)
10	133.2, C	-	-
11	122.4, C	-	-
12	157.3, C	-	-
13	116.1, CH	6.99, br. s	21.9 (18); 117.9 (2); 123.5 (15); 157.3 (12)
14	140.4, C	-	-
15	123.5, CH	7.53, br. s	21.9 (18); 116.1 (13); 166.5 (19)
16	133.2, C	-	-
17	56.3, CH_3_	3.77, s	157.3 (12)
18	21.9, CH_3_	2.44, s	123.5 (15); 116.1 (13); 140.4 (14)
19	166.5, C	-	-
20	61.1, CH_2_	4.17, (q, *J* = 7.1 Hz)	14.2 (21); 166.5 (19)
21	14.2, CH_3_	1.19 (t, *J* = 7.1 Hz)	61.1 (20)

**Table 2:** Effects of compounds **1–5** (WS-5995 A–E) on cancer cell proliferation.

Compounds	IC_50_ (μM) for 72 h					
	MCF-7	BT-549	RBE	A549	NCM-460	Huvec
**1**	>50	>50	>50	>50	>50	>50
**2**	>50	>50	21.39	>50	40.84	31.23
**3**	19.60	5.97	16.79	>50	18.47	7.67
**4**	>50	>50	>50	>50	>50	>50
**5**	>50	>50	>50	>50	>50	>50
Adriamycin	2.96	6.42	3.62	4.62	3.24	3.87
Cisplatin	4.49	14.21	4.26	9.34	19.03	5.26

## Data Availability

The data presented in this study are available on request from the corresponding authors.

## Supplementary Materials

The following supporting information can be downloaded at: https://www.mdpi.com/article/10.3390/md22050195/s1, Table S1: Strains and plasmids used or constructed in this study; Table S2: Primers used in this study; Table S3: Media used in this study; Table S4: antiSMASH prediction of biosynthetic gene clusters in *Streptomyces* sp. S1502; Table S5: Deduced functions of ORFs of *wsm* gene cluster in *Streptomyces* sp. S1502; Figure S1: Phylogenetic analysis of *Streptomyces* sp. S1502 using autoMLST; Figure S2: Disruption of *stp1* in wild type *S.* sp. S1502 via PCR-targeting; Figure S3: HR-ESI-MS spectrum of WS-5995 E (**1**); Figure S4–S8: NMR spectrum of WS-5995 E (**1**); Figure S9–S12: HR-ESI-MS spectrum of WS-5995 A–D (**2**–**5**); Figure S13–S20: NMR spectrum of WS-5995 A–D (**2**–**5**); Figure S21: Similar gene cluster to *wsm* in antiSMASH database; Figure S22: Disruption of *wsmA* in *S.* sp. S1502/Δ*stp1* via PCR-targeting; Figure S23: Amino acid sequence alignment of JadG, WsmI, GilOII and AlpJ; Figure S24: transformation of WS-5995 C; Figure S25: Comparison of *wsm* and *wsd* gene clusters using Clinker; Figure S26: PCR verification of disruption of *genes of wsm* in *S.* sp. S1502/Δ*stp1* via modified PCR-targeting [33,34].
