# Supplementary material for: Metabolic Blockade-Based Genome Mining of Sea Anemone-Associated Streptomyces sp. S1502 Identifies Atypical Angucyclines WS-5995 A–E: Isolation, Identification, Biosynthetic Investigation, and Bioactivities
Source: Mar Drugs. 2024 Apr 25;22(5):195. doi: 10.3390/md22050195 (PMC11122949; doi:10.3390/md22050195)
Supplement: Supplementary file 1 [file marinedrugs-22-00195-s001.zip › marinedrugs-2960565-supplementary.pdf]

Supporting information

Metabolic Blockade-Based Genome Mining of Sea Anemone-Associated *Streptomyces* sp. S1502 Identifies Atypical angucyclines WS-5995A-E: Isolation, Identification, Biosynthetic Investigation and Bioactivities

Yuyang Wang [1,2], Le Zhou [1], Xiaoting Pan[3], Zhangjun Liao[4], Nanshan Qi[3], Mingfei Sun[3], Hua Zhang[4], Jianhua Ju [1,2], Junying Ma *[1,2]

[1] CAS Key Laboratory of Tropical Marine Bio-Resources and Ecology, RNAM Center for Marine Microbiology, Guangdong Key Laboratory of Marine Materia Medica, South China Sea Institute of Oceanology, Chinese Academy of Sciences, Haizhu District, Guangzhou, Guangdong 510301, China

[2] College of Oceanology, University of Chinese Academy of Sciences, Qingdao 266400, China

[3] Key Laboratory of Livestock Disease Prevention of Guangdong Province, Key Laboratory of Avian Influenza and Other Major Poultry Diseases Prevention and Control, Ministry of Agriculture and Rural Affairs, Institute of Animal Health, Guangdong Academy of Agricultural Sciences, Guangzhou, 510640, China.

[4] Guangdong Provincial Key Laboratory of Medical Molecular Diagnostics, Institute of Laboratory Medicine, Guangdong Medical University, Dongguan 523808, China

Table S1. Strains and plasmids used or constructed in this study

Strains or plasmids	Characterization	Reference or source
**Strains**		
E. coli BW25113/pIJ790	Host strain for homologous reorganization during PCR-targeting process	[1]
E. coli DH5α/pIJ773	Host strain of plasmid pIJ773	[1]
E. coli ET12567/pUZ8002	Donor strain for conjugation with *Streptomyces*	[1]
E. coli ET12567/pUB307	Donor strain for conjugation with *Streptomyces*	[1]
Streptomyces sp. S1502	The wide-type strain, producer of streptopyrroles	This study
Δ*stp1*	*stp1* gene deletion mutant	This study
Δ*stp1*/Δ*wsmA*	*wsmA* gene in-frame deletion mutant	This study
Δ*wsmO/P/Q/R/*O_3*/X/ Y/*R_1*/*R_2*/*R_3*/*R_4*/XY*	Δ*wsmO/P/Q/R/*O_3*/X/ Y/*R_1*/*R_2*/*R_3*/*R_4*/XY* gene deletion mutant	This study
S. atratus SCSIO ZH16NSEP	Derived from *S. atratus* SCSIO ZH16; host strain for heterologous expression	This study
S. lividans SBT5	Host strain for heterologous expression	This study
**Plasmids**		
SuperCosI	Amp^r, Kan^r, cosmid vector	
pIJ790	Cm^r, including λ-RED (*gam*, *bet*, *exo*) for PCR-targeting	[1]
pIJ773	Apr^r, source of *acc(3)IV* and *oriT* fragment	[1]
pUZ8002	Kan^r, including *tra* for conjugation	[1]
pUB307	Apr^r, including *tra* for conjugation	[2]
Cosmid 10-11F	Amp^r, Kan^r, a cosmid which contains partial streptopyrrole biosynthetic gene cluster	This study
Cosmid 13-4D; 13-5D	Amp^r, Kan^r, a cosmid which contains Δ*ilaMN* deleted partial WS-5995 biosynthetic gene cluster	This study
pBAC/1-9A	a cosmid which contains intact *wsm* biosynthesis cluster	This study

Abbreviations: Amp^r, ampicillin resistance; Kan^r, kanamycin resistance; Apr^r, apramycin

resistance, Cm^{r}, chloramphenicol resistance

Table S2. Primers used in this study

Name	Sequence (5′-3′)	Purpose
**For screening the cosmid library and BAC library**		
Orf-wsmAa-scF	CGGATCTACCTCGGTGCGG	For screening the cosmid library and BAC library; and for verifying right exconjugants
Orf-wsmAa-scR	GGTAGATGTTGCCGTGGGCC	
Orf-wsmAb-scF	GGGGCTCGAGGTTCTCGCAC	
Orf-wsmAb-scR	CCGGCTCGGTCAGCACCATC	
Orf-wsmAc-scF	GAGTTCACCGGGCTGGAGGC	
Orf-wsmAc-scR	GGTTGACGGCGTCCAGGATG	
Orf-wsmAd-scF	GCTAGGGCTCATCACGTTCG	
Orf-wsmAd-scR	CGGCTGTACGGTGACTTCGG	
Orf-wsmAe-scF	GCATCGAGCGGGACCTGCAC	
Orf-wsmAd-scR	CCTGAGGGGCTGTGGACGTG	
**For gene replacement**		
Del_stp1-F	CCGGACGACCGGCTCTCCAACCACGCGCCGT TCAACTTCactagtATTCCGGGGATCCGTCGACC	For in-frame disrupting *stp1*
Del_ stp1-R	GACGCAGACGGAAACCCGGTCGACGATCATG TAGGTGGGactagtTGTAGGCTGGAGCTGCTTC	
Del_*wsmA*-F	GCCAAGAACTTCTGGAGCCTGCTGAGTGAGG GACGCACGactagtATTCCGGGGATCCGTCGACC	For in-frame disrupting *wsmA*
Del_*wsmA*-R	GCTGCCGACGGTGAGTACCGTGTCGGTGAGCT GGTCGCGactagtTGTAGGCTGGAGCTGCTTC	
del_*wsmX*-F	GCCTGGTACACCGTCGGCGAGCACCACTTC GGTGAGCGGATTCCGGGGATCCGTCGACC	For disrupting *wsmX* and *wsmXY*
del_*wsmX*-R	CAGGTGCAGGTCGTGGCCCCACAGCTCGTG CAGGCGCCCTGTAGGCTGGAGCTGCTTC	
del_*wsmY*-F	GTCTGGGTCGACGTCGACACGGTCCTGTCC GCCCCGCCCATTCCGGGGATCCGTCGACC	For disrupting *wsmY* and *wsmXY*

del_*wsmY*-R	GCAGGCGGTGACTCCGCTGCCGCAGTAGAC<br>GACGACGTCTGTAGGCTGGAGCTGCTTC	
del_$wsmO_3$-F	GCGGTGATCGCCGCGTCCACCACGCGTGTA<br>CGGGTCTTCATTCCGGGGATCCGTCGACC	For disrupting $wsmO_3$
del_$wsmO_3$-R	CAGGCTCAGCCGCTGGATCTCCGCTCGCGC<br>GTCCTGTAGGCTGGAGCTGCTTC	
del_$wsmR_1$-F	CTGCCGTCGTACCTCGCCGGGCACGTGGCG<br>CGCATCGGCATTCCGGGGATCCGTCGACC	For disrupting $wsmR_1$
del_$wsmR_1$-R	CCGTTCCTCGGGGGTGAAGCAGCCGAGGA<br>AGGAGTCCTGTGTAGGCTGGAGCTGCTTC	
del_$wsmR_2$-F	GACGGCCTCGAGGTGTGCAAGGCCATCCGC<br>GCCGTCAGCATTCCGGGGATCCGTCGACC	For disrupting $wsmR_2$
del_$wsmR_2$-R	GCGGAGGCTGCTGACGTGGGTGTCGACGGT<br>CCGCCGGGATGTAGGCTGGAGCTGCTTC	
del_*wsmO*-F	CCCTTTCCCGGCAAGGTGGTGATCCGGTCG<br>GTGGCGCTCATTCCGGGGATCCGTCGACC	For disrupting *wsmO*
del_*wsmO*-R	GGCGTAGTAGCCGTCGGGGCGGATCAGGAG<br>CGCCTCGTCTGTAGGCTGGAGCTGCTTC	
del_*wsmP*-F	GCGGACGTCGCGGACGACACGGCGAAGGC<br>GGCCGCCGAGATTCCGGGGATCCGTCGACC	For disrupting *wsmP*
del_*wsmP*-R	CTGGCTCACGTCCTGCGGAGTACCGAGGCG<br>CGGGATCAGTGTAGGCTGGAGCTGCTTC	
del_*wsmQ*-F	GCGGCCCTGCGCGGGCTGGTCGACGAGGA<br>ATGGGCCCGGATTCCGGGGATCCGTCGACC	For disrupting *wsmQ*
del_*wsmQ*-R	CGAGGCACCGCCGGTCGAGCCCAGCGGCA<br>CGAGCACCGCTGTAGGCTGGAGCTGCTTC	
del_*wsmR*-F	GGGGTCAACCCGGTCGACTGGAAACGCCGT<br>TCGGGCCGCATTCCGGGGATCCGTCGACC	For disrupting *wsmR*

del_*wsmR*-R	CACGACCGCGCCCAGGATCGCCGGGTCGCG CACCGGCCCTGTAGGCTGGAGCTGCTTC	
del_$wsmR_3$-F	GCGAAACGCCGGGCCATCACCCAAGGCGC GCGTGCCGTCATTCCGGGGATCCGTCGACC	For disrupting
del_$wsmR_3$-R	GACGAAGTGGAGGGTGGCCCGGGCGGGGC TGGAGATGTGTGTAGGCTGGAGCTGCTTC	$wsmR_3$
del-$wsmR_4$-F	GACAAGGGCTACGACGCCATGACCGTCGGC GACATCGCCATTCCGGGGATCCGTCGACC	For disrupting
del-$wsmR_4$-R	GGCGTGGTAGAAGGTCCGTTCGATCATCCA GCACAGGGCTGTAGGCTGGAGCTGCTTC	$wsmR_4$
**For verifying the mutants**		
ID_stp1-F	GAACCTCCGACATCCAGCGC	For verifying the
ID_ stp1-R	TGACCAGCCCACGCCTG	disruption of Δ*stp1*
ID-wsmX-F	GCTAGGGCTCATCACGTTCG	For verifying
ID-wsmX-R	GCGGCGGTCAGTTCGGTG	Δ*wsmX* and Δ*wsmXY*
ID-wsmY-F	GACGCCGGATGGGTGAGCG	For verifying
ID-wsmY-R	GGACAGGTCCGTGGACCAGG	Δ*wsmY* and Δ*wsmXY*
ID-$wsmR_4$-F	GGCCACCACCCCGCACGAAG	For verifying
ID-$wsmR_4$-R	GATGTGTTCGCATGTCGCCG	Δ$wsmR_4$
ID-$wsmO_3$-F	CGTGCCGGACCACCCCTAC	For verifying
ID-$wsmO_3$-R	CAGCACCTGGATGACGTGGTG	Δ$wsmO_3$
ID-$wsmR_1$-F	CGGTCCGCTGTTTCGGAGC	For verifying
ID-$wsmR_1$-R	GTGGTCACTCGGCGCACG	Δ$wsmR_1$
ID-$wsmR_2$-F	GCGTGGACCTCGTCCTGATGG	For verifying
ID-$wsmR_2$-R	GCCGCGTACGGTGACGACC	Δ$wsmR_2$
ID-*wsmO*-F	CGAAGGGCATCAGCAACCTG	For verifying
ID-*wsmO*-R	GGGATGGTCTGCGTTCGGCC	Δ*wsmO*

ID-*wsmP*-F	GTCGAACGGAACGCAGTCCC	For verifying
ID-*wsmP*-R	GGCCGAACGCAGACCATCCC	Δ*wsmP*
ID-*wsmQ*-F	CGGATACGGAGGGTCTGCAC	For verifying
ID-*wsmQ*-R	CACGGGGCTTGTGGAAGGTC	Δ*wsmQ*
ID-*wsmR*-F	GCCAGACAGAGACGTCGAGG	For verifying
ID-*wsmR*-R	GCCCGCTCGAACGGGATCAC	Δ*wsmR*
ID-*wsmR*$_3$-F	GCTGCATGGCAATTCGGCCG	For verifying
ID-*wsmR*$_3$-R	GGAGCGCGGCTGGTGTAGG	Δ*wsmR*$_4$
ID-*wsmR*$_4$-F	GGCCACCACCCCGCACGAAG	For verifying
ID-*wsmR*$_4$-R	GATGTGTTCGCATGTCGCCG	Δ*wsmR*$_{44}$

Table S3. Media used in this study

Medium name	Ingredient
ISP-4	1% soluble starch, 0.1% bacteria peptone, 0.05% yeast extract, 0.1% K_2HPO_4, 0.1% $MgSO_4·7H_2O$, 0.2% $(NH_4)_2SO_4$, 0.1% NaCl, 0.01% trace element solution, 3% artificial sea salt, 2% $CaCO_3$, pH 7.2–7.4
MS	2% mannitol, 2% soybean powder, 1.5% agar, 3% artificial sea salt, and 0.2% $CaCO_3$, pH 7.2–7.4
RA	2% soluble starch, 0.5% corn flour, 1% malt extract, 1% glucose, 1% maltose, 0.01% trace elements, 3% artificial sea salt, and 0.2% $CaCO_3$, pH 7.2–7.4 (3% XAD-16 resins)
LB	0.5% yeast extract, 1% Tryptone, 1% NaCl, pH 7.2–7.4

Table S4. antiSMASH prediction of biosynthetic gene clusters in *Streptomyces* sp. S1502.

BGC	BGC type	Compounds	Similarity (%)
1	T1PKS	limazepine	11
2	NRPS	coelibactin	100
3	indole	7-prenylisatin	33
4	terpene	isorenieratene	62
5	NRPS	paenibactin	83
6	T3PKS	germicidin	100
7	T1PKS	sceliphrolactam	56
8	T3PKS	herboxidiene	8
9	ectoine	ectoine	100
10	melanin	melanin	60
11	T1PKS	pyrrolomycin	37
12	siderophore	desferrioxamin	83
13	RRE-containing	naphthomycin A	9
14	terpene	albaflavenone	100
15	T2PKS	spore pigment	66
16	siderophore	-	-
17	T2PKS; NRPS	WS-5995/friulimicin	69/75
18	Ripp-like	-	-
19	terpene	geosmin	100
20	siderophore	-	-
21	NRPS; T1PKS	α-lipomycin	100
22	PKS	leinamycin	15
23	lanthipeptide-class-iii	SapB	100
24	terpene	hopene	100
25	Ripp-like	informatipeptin	42
26	NRPS	coelichelin	100
27	ranthipeptide	-	-

Table S5. Deduced functions of ORFs of *wsm* gene cluster in *Streptomyces* sp. SCSIO S1502.

ORF	size[a]	Proposed function	Protein homologue and origin	ID/SI[b]
wsmR$_4$	202	TetR family transcriptional regulator	ARP51756.1 (*Streptomyces* sp. SCSIO 03032)	39/67
wsmO$_1$	280	KR; short-chain dehydrogenase/reductase SDR	TtmK (*Streptomyces afghaniensis*)	42/53
wsmU	107	Polyketide synthesis cyclase	WsdU (*Streptomyces yanglinensis* CGMCC 4.2023)	77/83
wsmA	421	Beta-ketoacyl synthase	WsdA (*Streptomyces yanglinensis* CGMCC 4.2023)	84/89
wsmB	405	Beta-ketoacyl synthase/CLF	WsdB (*Streptomyces yanglinensis* CGMCC 4.2023)	74/82
wsmC	87	ACP	WsdC (*Streptomyces yanglinensis* CGMCC 4.2023)	65/75
wsmD	260	KR; C9	WsdD (*Streptomyces yanglinensis* CGMCC 4.2023)	80/86
wsmE	313	CYC C7-C12	WsdE (*Streptomyces yanglinensis* CGMCC 4.2023)	67/76
wsmF	442	monooxygenase FAD-binding	WsdF (*Streptomyces yanglinensis* CGMCC 4.2023)	55/62
wsmI	227	putative_anthrone_monooxygenase	WsdI (*Streptomyces yanglinensis* CGMCC 4.2023)/JadG/GilOII	69/81 (71/85)
wsmO$_2$	494	monooxygenase FAD-binding	WsdT (*Streptomyces yanglinensis* CGMCC 4.2023)	59/69
wsmG	148	nuclear transport factor 2 family protein	WsdG (*Streptomyces yanglinensis* CGMCC 4.2023)	40/53
wsmH	338	*O*-methyltransferase	WsdH (*Streptomyces yanglinensis* CGMCC 4.2023)	65/76
wsmJ	302	oxidoreductase	WsdJ (*Streptomyces yanglinensis*	60/73

			CGMCC 4.2023)	
wsmK	531	extracellular solute-binding protein family 5	WsdK (*Streptomyces yanglinensis* CGMCC 4.2023)	37/50
wsmL	317	binding-protein-dependent transport systems	WsdL (*Streptomyces yanglinensis* CGMCC 4.2023)	44/60
wsmM	288	binding-protein-dependent transport systems	WsdM (*Streptomyces yanglinensis* CGMCC 4.2023)	41/58
wsmN	611	ABC transporter ATP-binding protein	WsdN (*Streptomyces yanglinensis* CGMCC 4.2023)	54/66
wsmX	347	luciferase family protein/LLM class flavin-dependent oxidoreductase	OvmZ (*Streptomyces olivaceus* SCSIO T05)	41/51
wsmY	268	putative rhodanese domain-containing protein	AWR88429.1 (*Streptomyces filamentosus*)	51/61
wsmO$_3$	538	oxidoreductase	HrbZ1 (*Streptomyces* sp. 2238-SVT4)	41/52
wsmZ	480	Drug resistance transporter, EmrB/QacA	BAB69179.1 (*Streptomyces avermitilis*)	42/60
wsmR$_1$	161	transcriptional regulator, MarR family	StfRIII (*Streptomyces steffisburgensis*)	39/50
wsmR$_2$	249	response regulator	WP_237282926.1 (*Streptomyces* sp. PKU-MA00045)	71/82
wsmO	476	monooxygenase FAD-binding	WsdO (*Streptomyces yanglinensis* CGMCC 4.2023)	77/83
wsmP	265	KR	WsdP (*Streptomyces yanglinensis* CGMCC 4.2023)	84/88
wsmQ	362	pyridine nucleotide-disulfide oxidoreductase	WsdQ (*Streptomyces yanglinensis* CGMCC 4.2023)	74/84
wsmR	300	crotonyl-CoA reductase / alcohol dehydrogenase	WsdR (*Streptomyces yanglinensis* CGMCC 4.2023)	82/86

wsmR$_3$	241	TetR family transcriptional regulator	BexR2 (*Amycolatopsis orientalis* subsp. *vinearia*)	48/59

a: amino acid length; b: identity/similarity

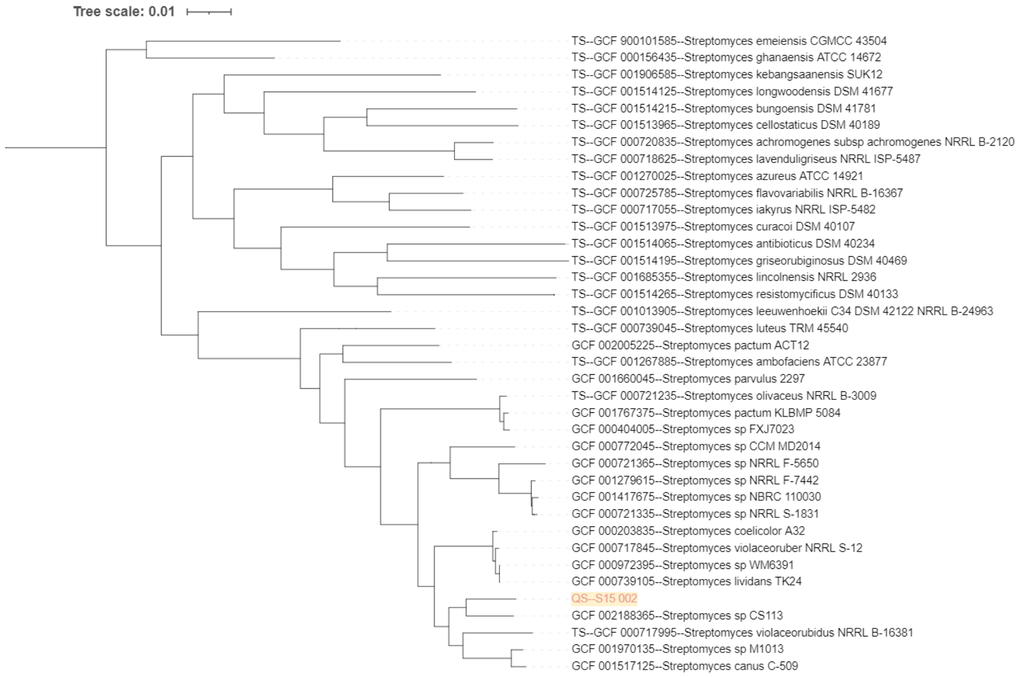

Figure S1 Phylogenetic analysis of *Streptomyces* sp. S1502 using autoMLST

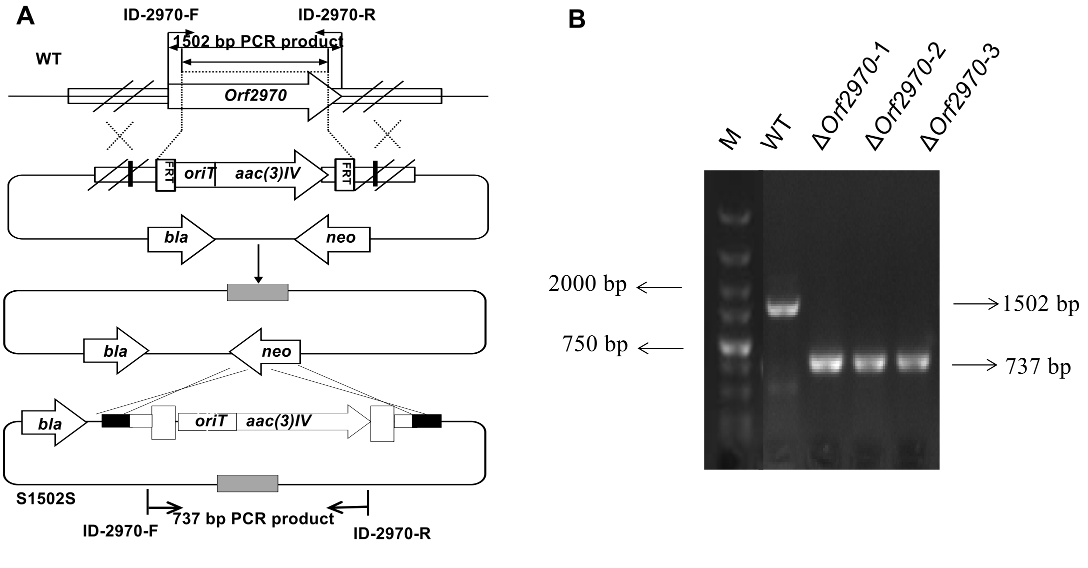

Figure S2. Disruption of *stp1* in wild type *S.* sp. S1502 via PCR-targeting. (A) Schematic representation for disruption of *stp1*. (B) PCR analyses of the WT strain and the *stp1* double-cross mutant carried out using the primers listed in Table S2. M: DNA molecular ladder; WT: using the genomic DNA of *S.* sp. S1502 as template; Δ*Orf2970*-1-3: using the genomic DNA of *stp1* mutant as template.

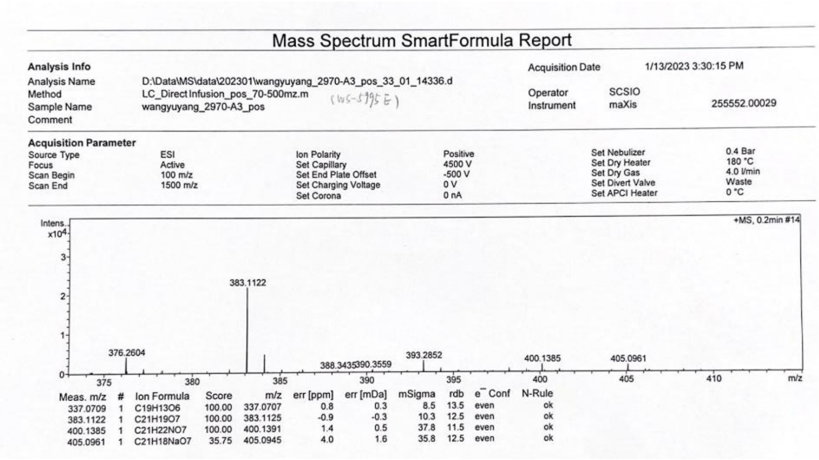

Mass Spectrum SmartFormula Report

**Analysis Info**		Acquisition Date	1/13/2023 3:30:15 PM
Analysis Name	D:\Data\MS\data\202301\wangyuyang_2970-A3_pos_33_01_14336.d		
Method	LC_Direct Infusion_pos_70-500mz.m (WS-5995 E)	Operator	SCSIO
Sample Name	wangyuyang_2970-A3_pos	Instrument	maXis 255552.00029
Comment			

**Acquisition Parameter**					
Source Type	ESI	Ion Polarity	Positive	Set Nebulizer	0.4 Bar
Focus	Active	Set Capillary	4500 V	Set Dry Heater	180 °C
Scan Begin	100 m/z	Set End Plate Offset	-500 V	Set Dry Gas	4.0 l/min
Scan End	1500 m/z	Set Charging Voltage	0 V	Set Divert Valve	Waste
		Set Corona	0 nA	Set APCI Heater	0 °C

Meas. m/z	#	Ion Formula	Score	m/z	err [ppm]	err [mDa]	mSigma	rdb	e⁻ Conf	N-Rule
337.0709	1	C19H13O6	100.00	337.0707	0.8	0.3	8.5	13.5	even	ok
383.1122	1	C21H19O7	100.00	383.1125	-0.9	-0.3	10.3	12.5	even	ok
400.1385	1	C21H22NO7	100.00	400.1391	1.4	0.5	37.8	11.5	even	ok
405.0961	1	C21H18NaO7	35.75	405.0945	4.0	1.6	35.8	12.5	even	ok

Figure S3. HE-ESI-MS spectrum of WS-5995 E (**1**)

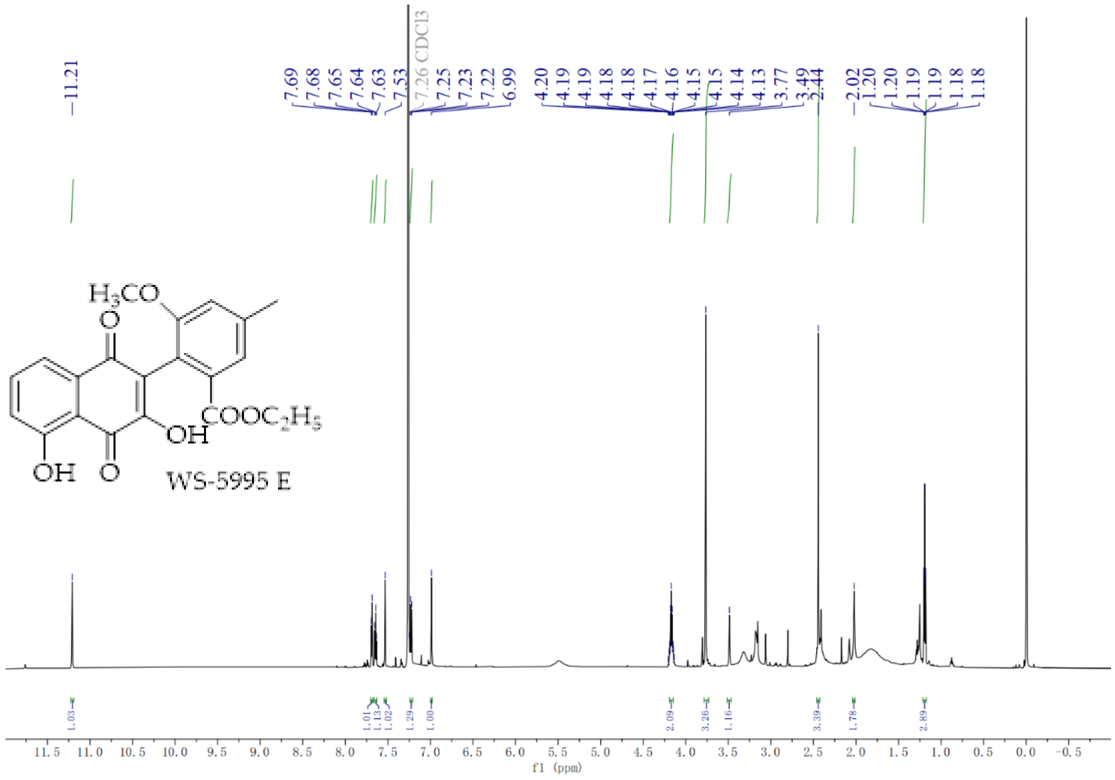

Figure S4. ^{1}H NMR (500 MHz, $CDCl_3$) spectrum of WS-5995 E (**1**).

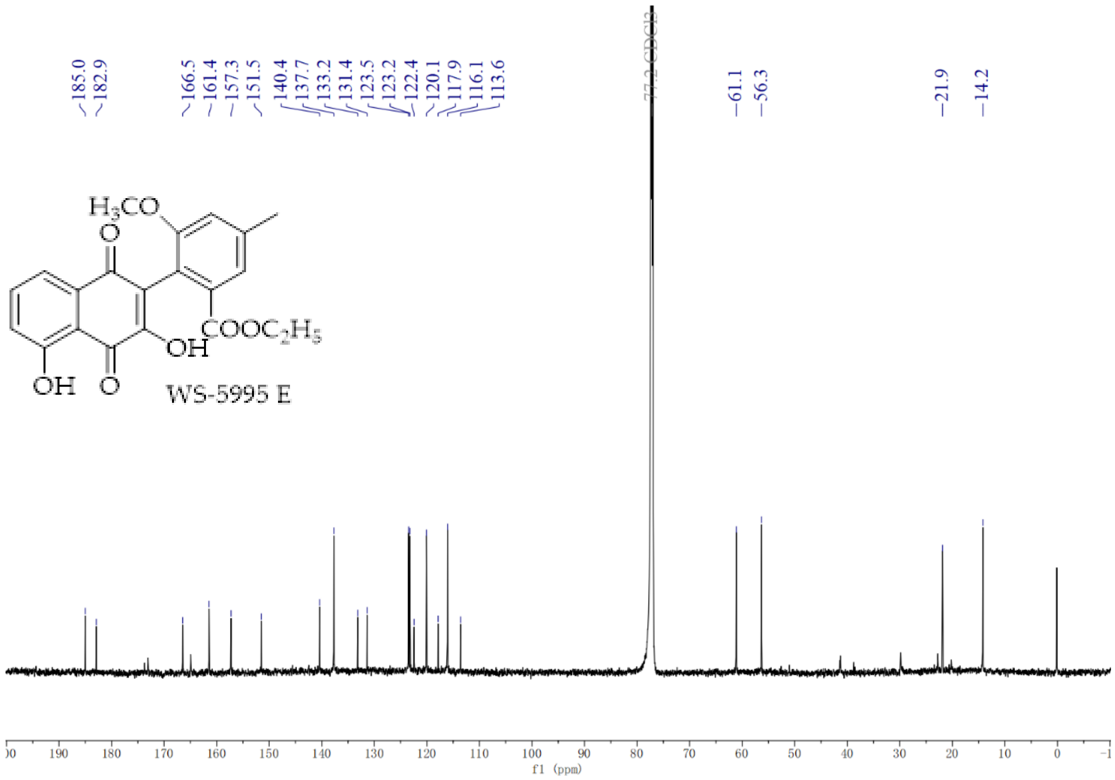

Figure S5. ^{13}C NMR (125 MHz, $CDCl_3$) spectrum of WS-5995 E (**1**).

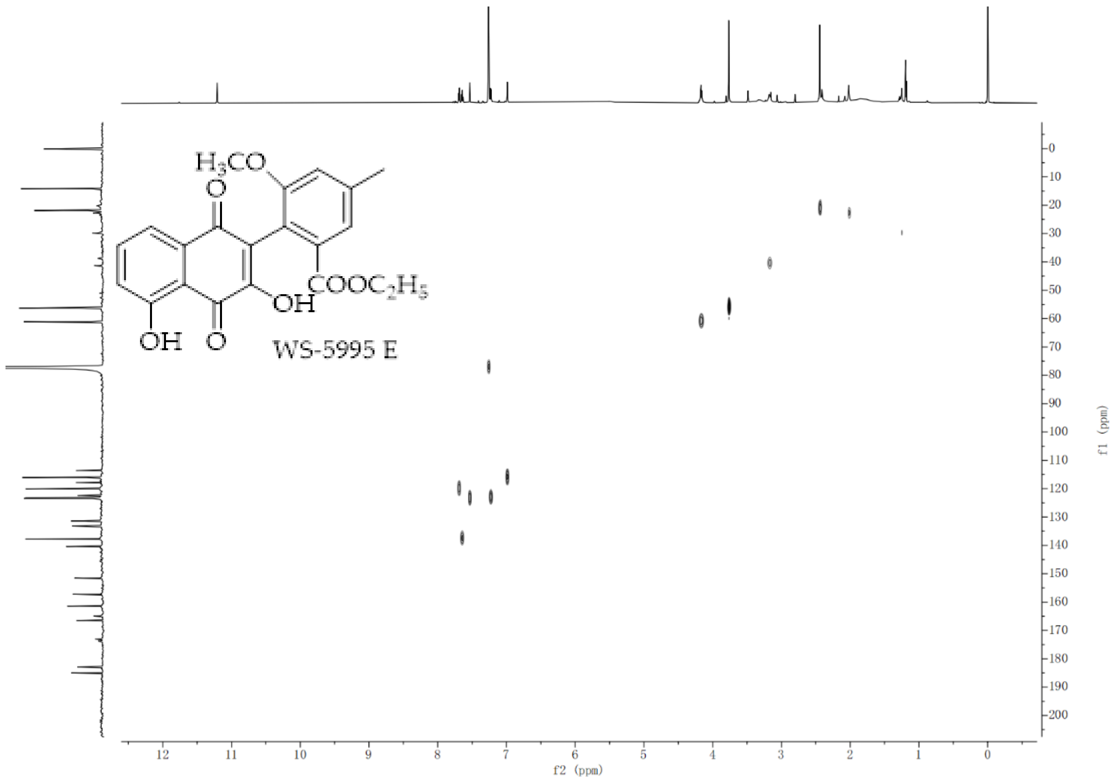

Figure S6. HMBC (500 MHz, $CDCl_3$) spectrum of WS-5995 E (**1**).

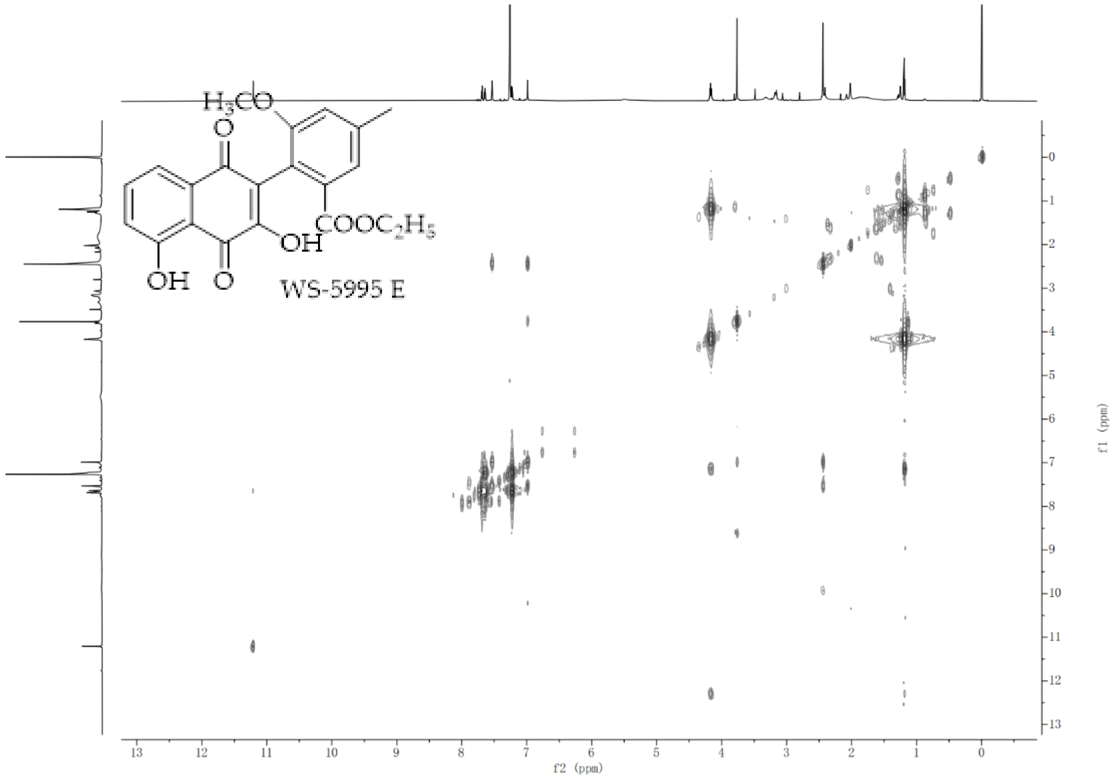

Figure S7. COSY (500 MHz, $CDCl_3$) spectrum of WS-5995 E (**1**).

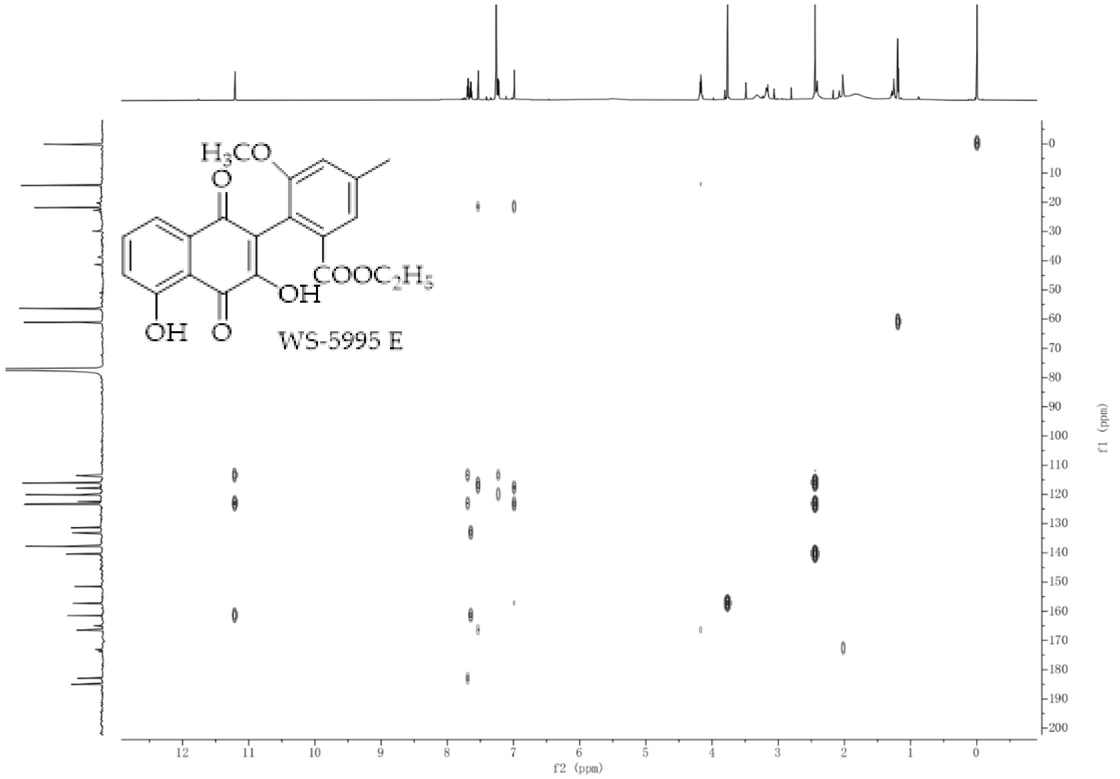

Figure S8. HSQC (500 MHz, $CDCl_3$) spectrum of WS-5995 E (**1**).

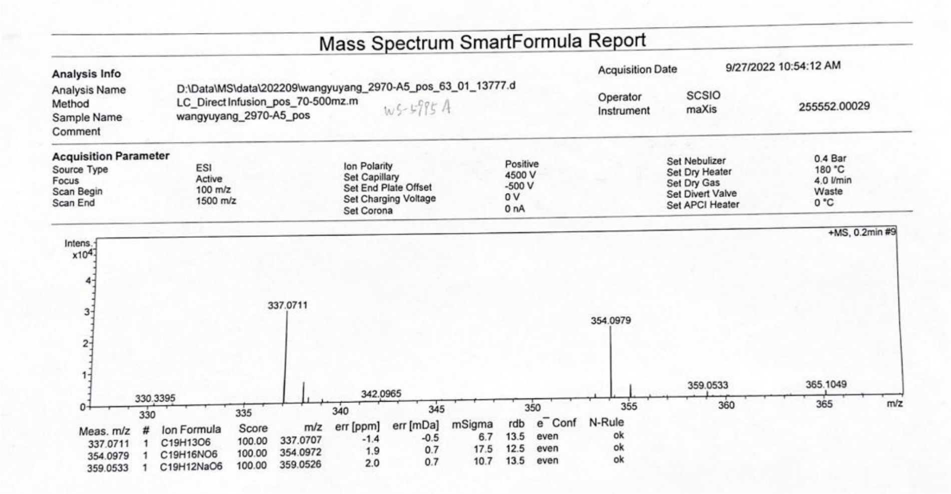

Figure S9. HE-ESI-MS spectrum of WS-5995 A (**2**)

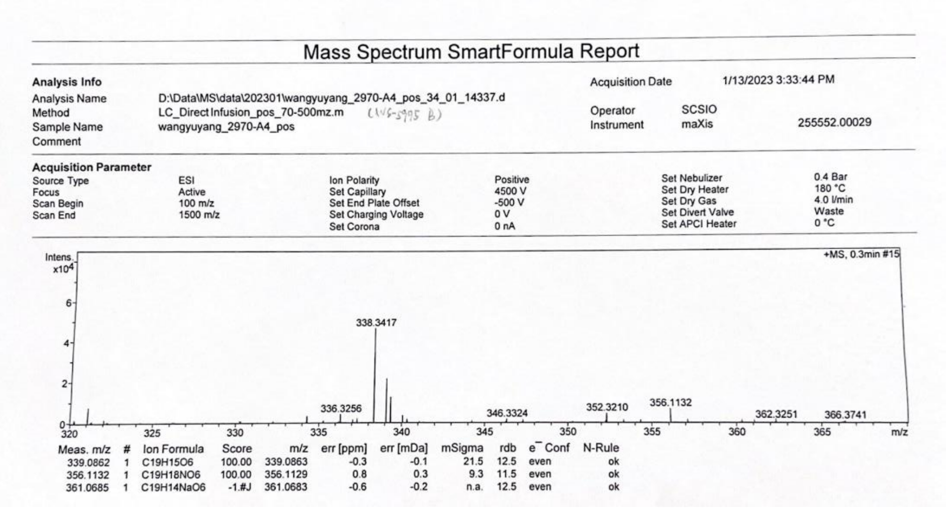

Figure S10. HE-ESI-MS spectrum of WS-5995 B (**3**)

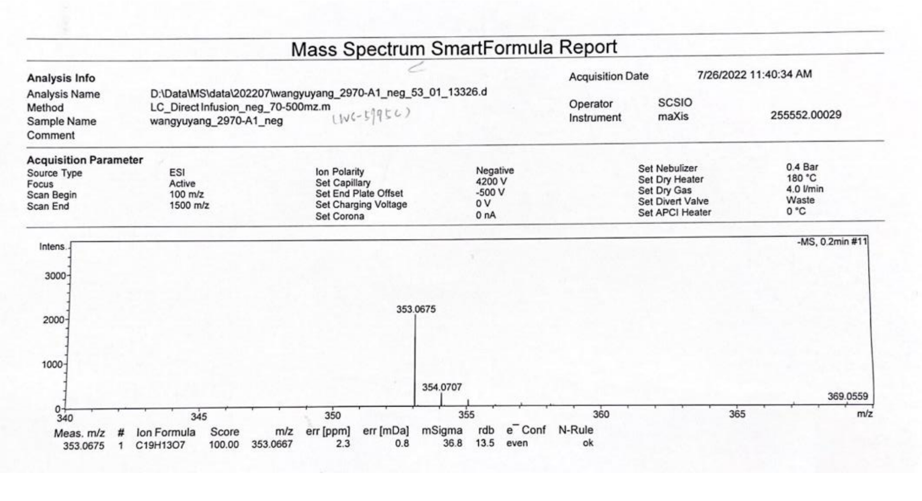

Figure S11. HE-ESI-MS spectrum of WS-5995 C (**4**)

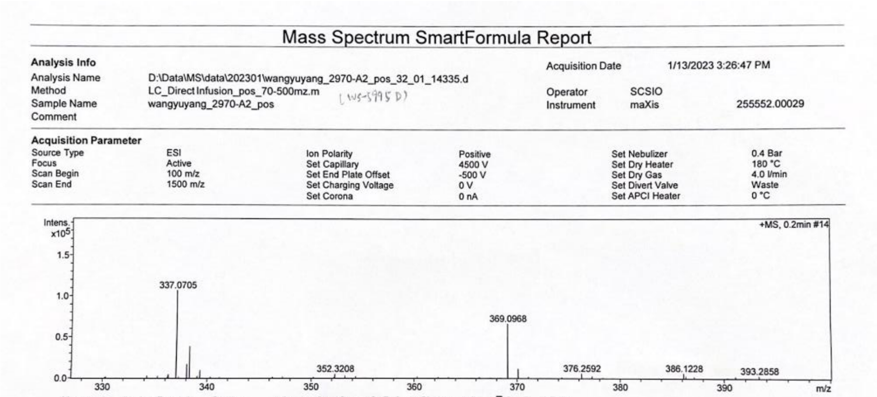

Meas. m/z	#	Ion Formula	Score	m/z	err [ppm]	err [mDa]	mSigma	rdb	e⁻ Conf	N-Rule
337.0705	1	C19H13O6	100.00	337.0707	0.5	0.2	24.7	13.5	even	ok
369.0968	1	C20H17O7	100.00	369.0969	0.3	0.1	21.7	12.5	even	ok
386.1228	1	C20H20NO7	100.00	386.1234	1.7	0.7	39.2	11.5	even	ok
391.0783	1	C20H16NaO7	100.00	391.0788	-1.3	-0.5	12.0	12.5	even	ok

Figure S12. HE-ESI-MS spectrum of WS-5995 D (**5**)

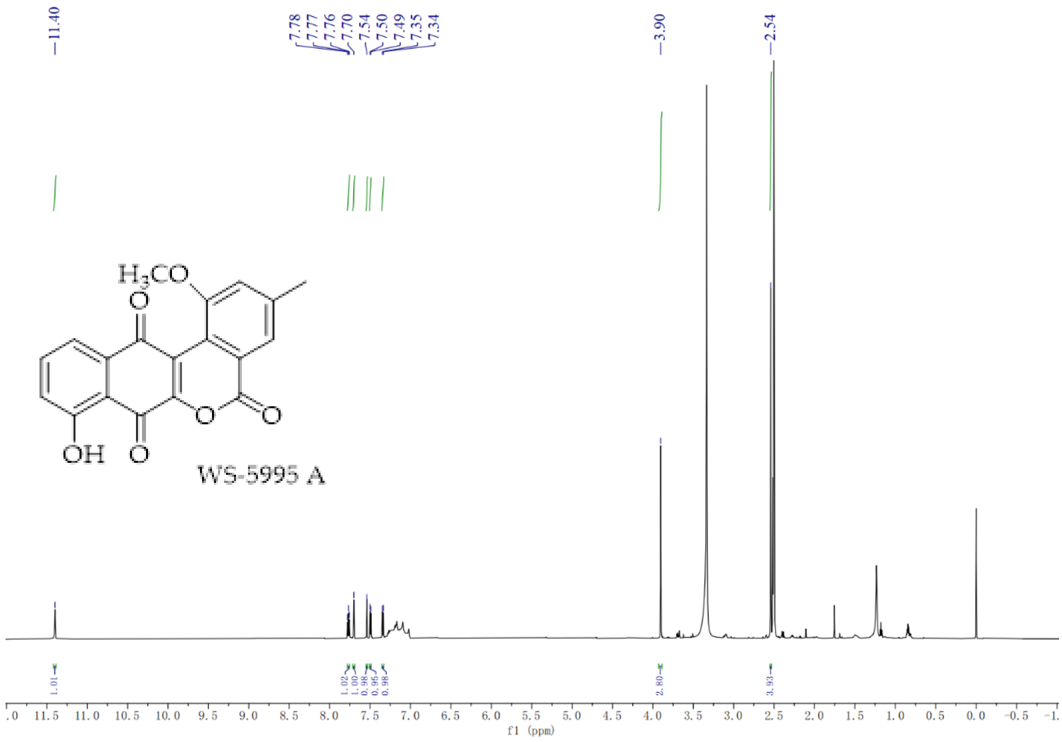

Figure S13. ^{1}H NMR (700 MHz, DMSO-d_6) spectrum of WS-5995 A (**2**).

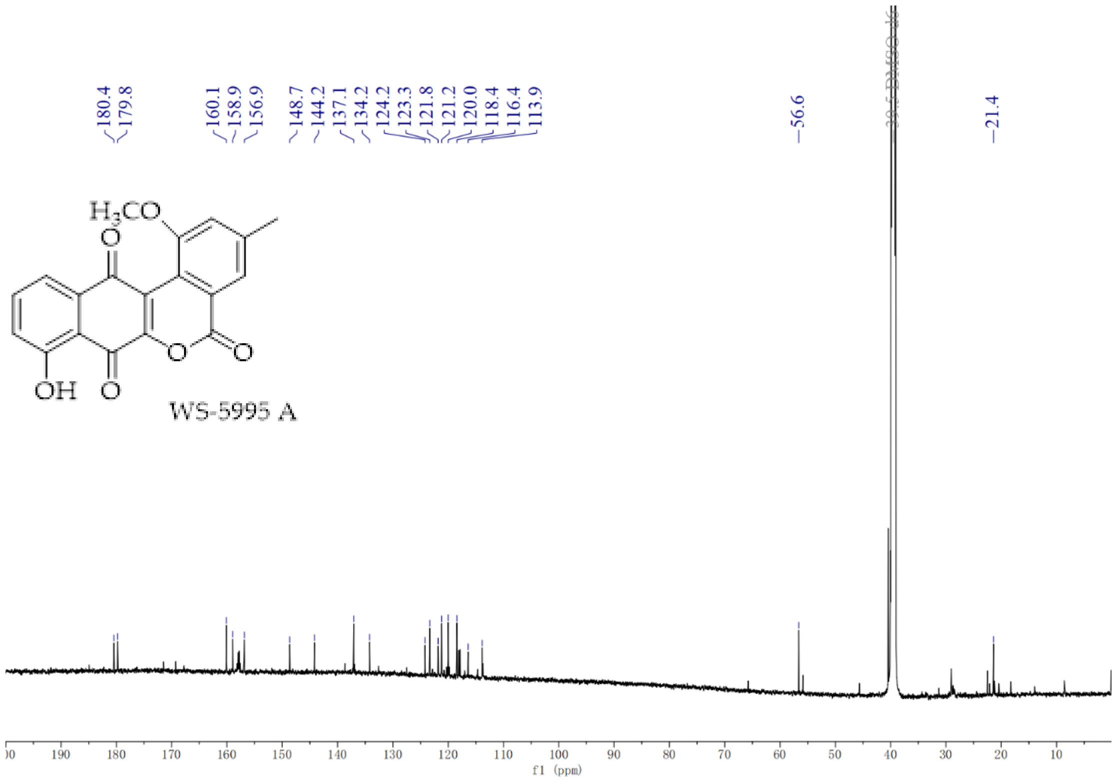

Figure S14. ^{13}C NMR (175 MHz, DMSO-d_6) spectrum of WS-5995 A (**2**).

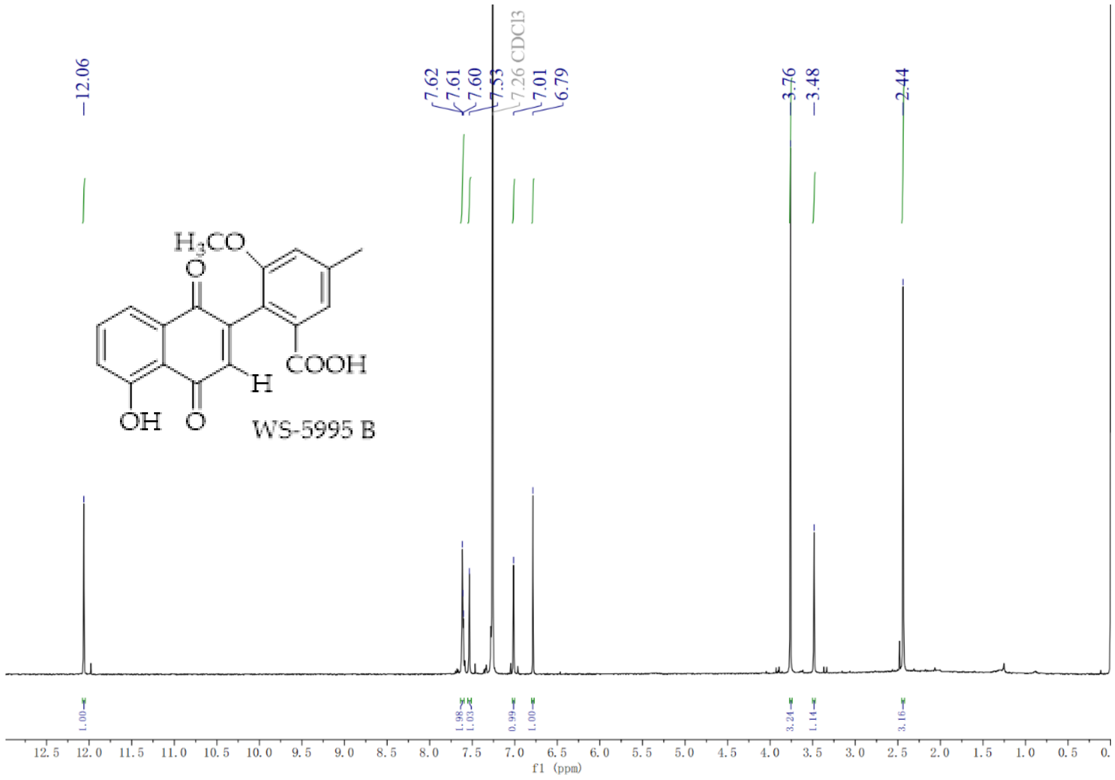

Figure S15. ^{1}H NMR (700 MHz, CDCl$_3$) spectrum of WS-5995 B (**3**).

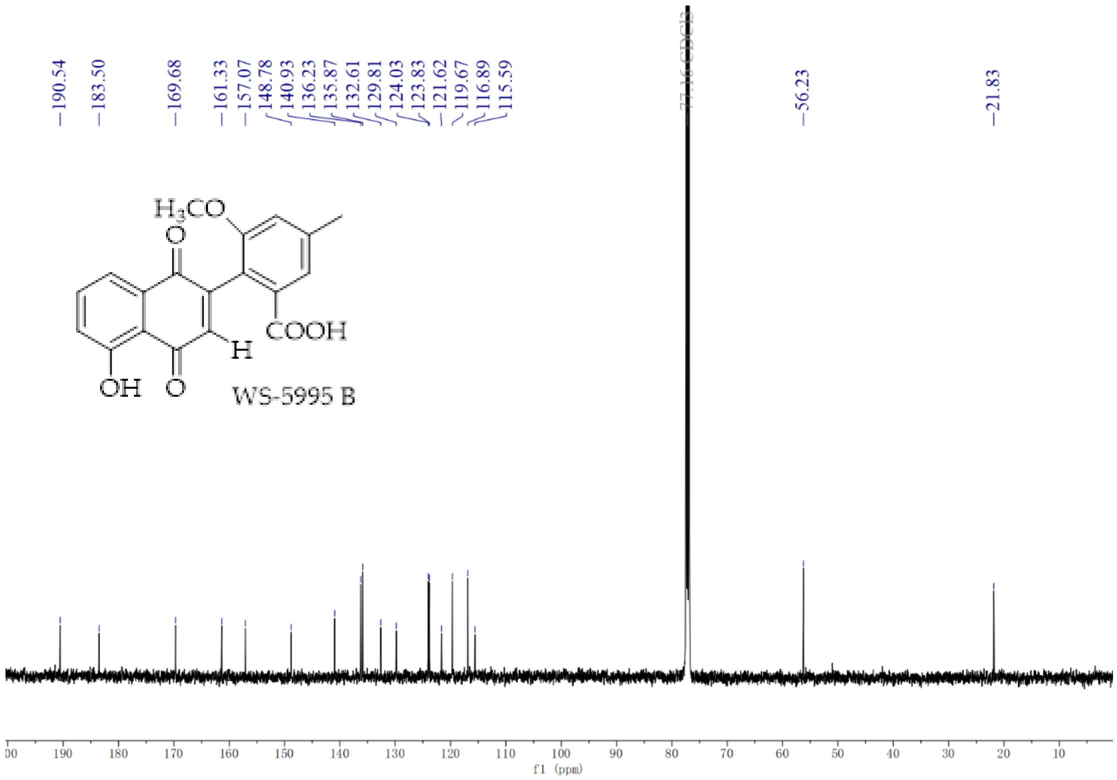

Figure S16. ^{13}C NMR (175 MHz, $CDCl_3$) spectrum of WS-5995 B (**3**).

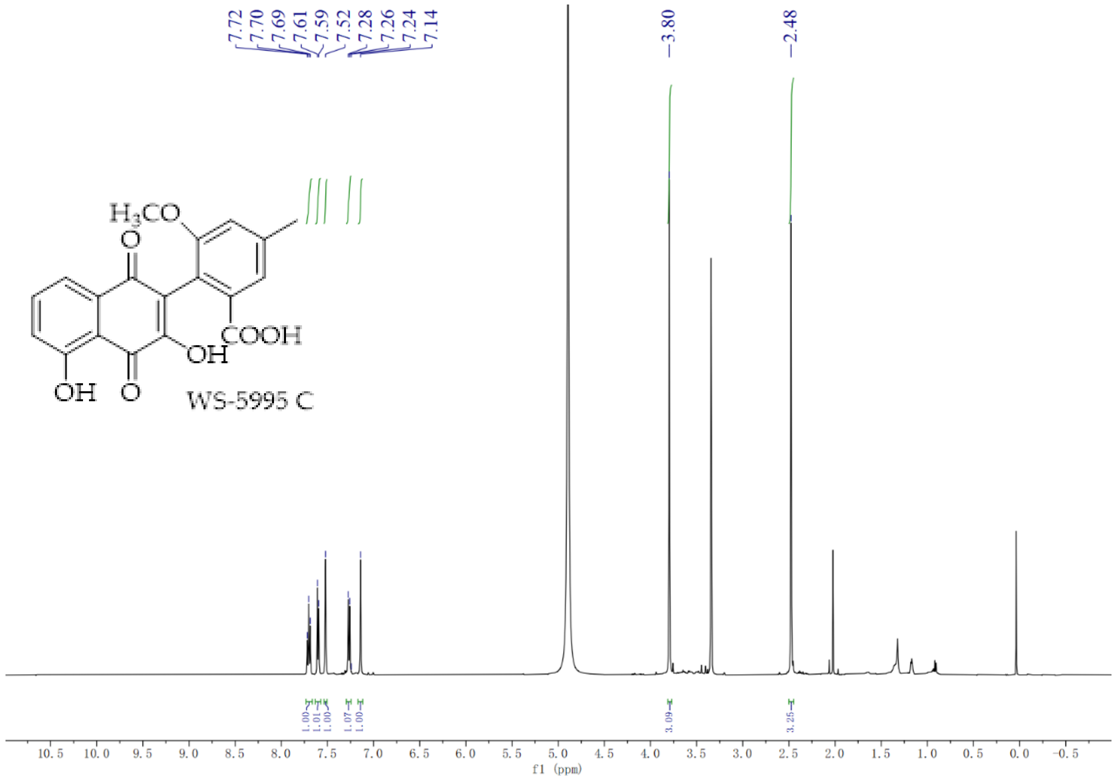

Figure S17. ^{1}H NMR (500 MHz, CD_3OD) spectrum of WS-5995 C (**4**).

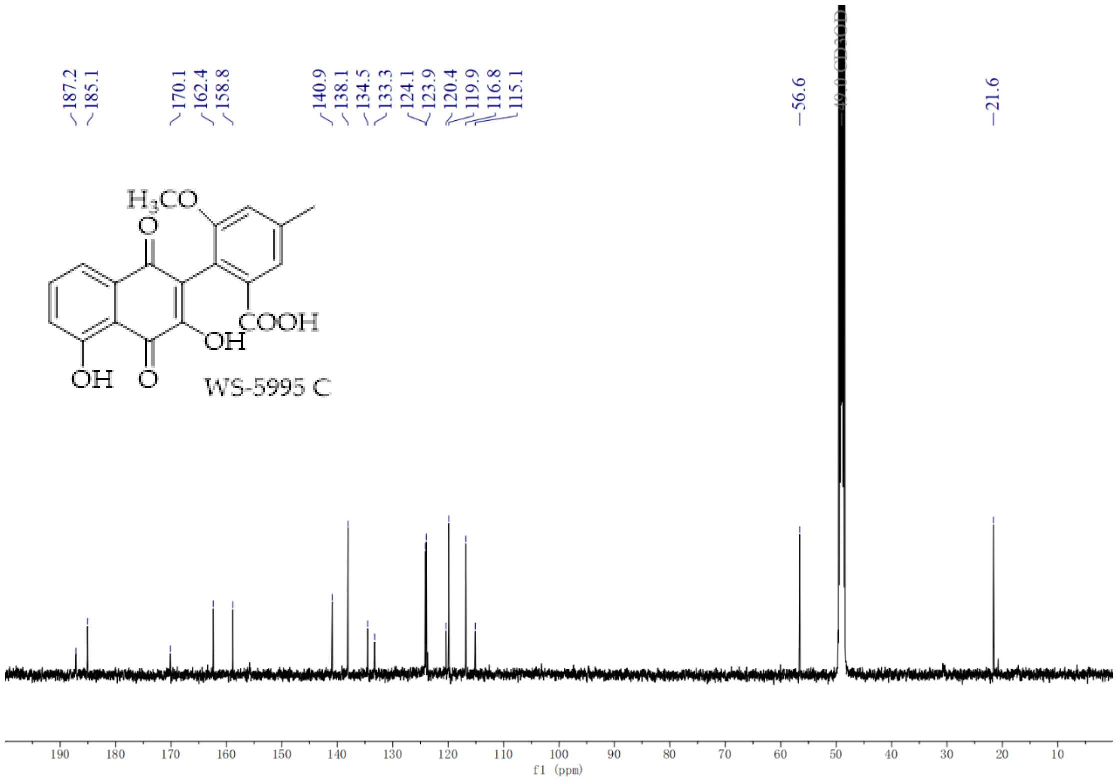

Figure S18. ^{13}C NMR (125 MHz, CD_3OD) spectrum of WS-5995 C (**4**).

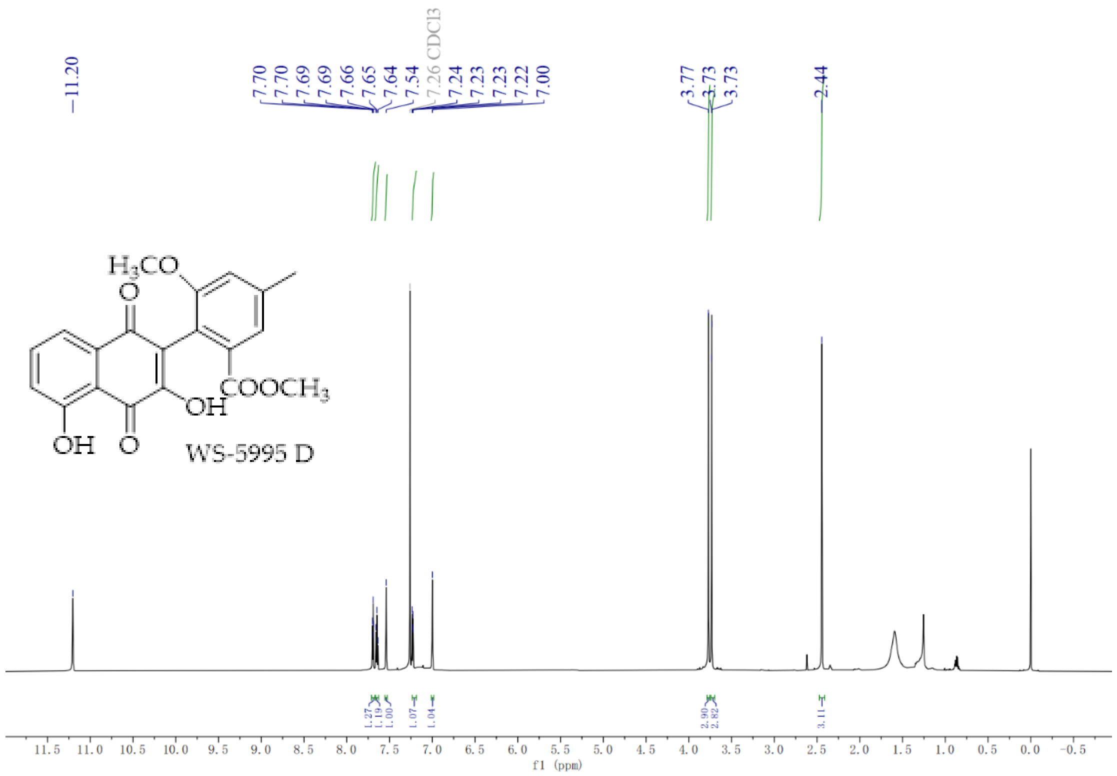

Figure S19. ^{1}H NMR (500 MHz, $CDCl_3$) spectrum of WS-5995 D (**5**).

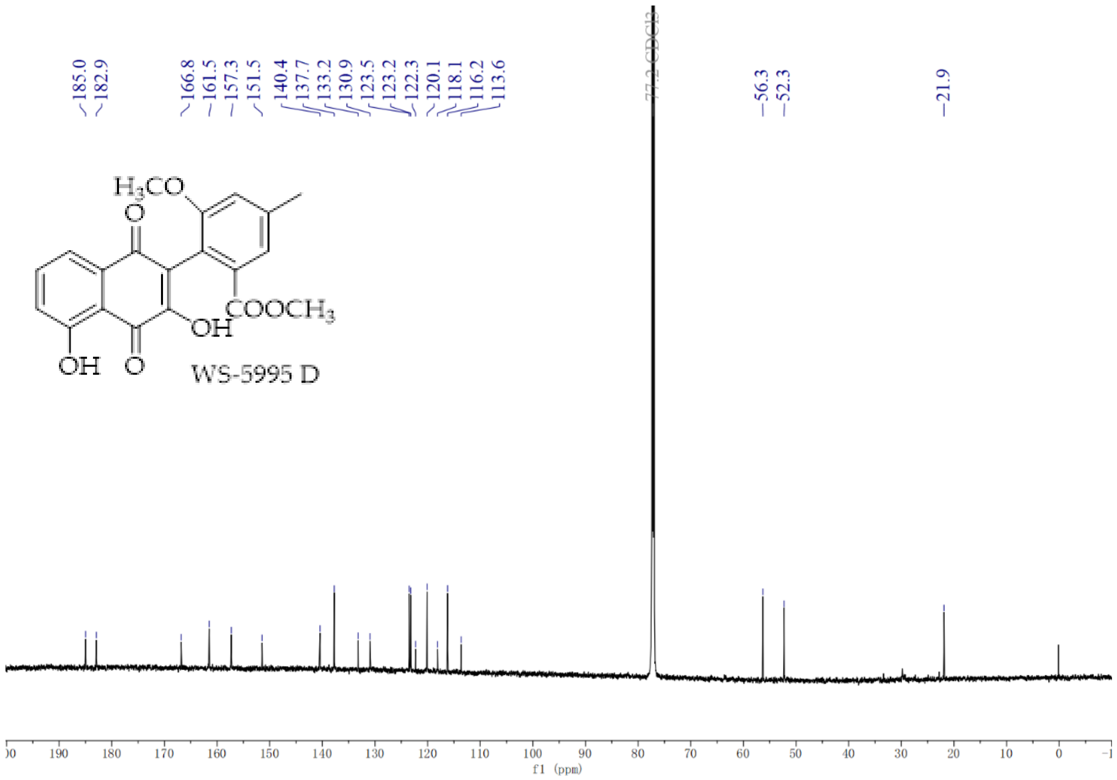

Figure S20. ^{13}C NMR (125 MHz, $CDCl_3$) spectrum of WS-5995 D (**5**).

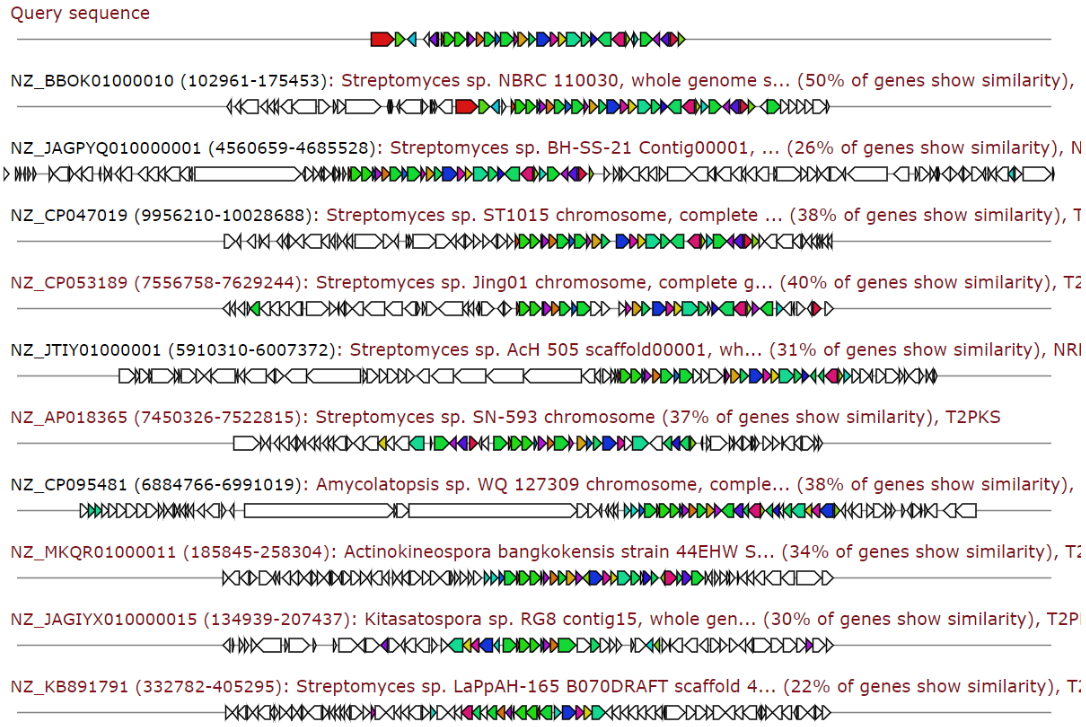

Figure S21 Similar gene cluster to *wsm* in antiSMASH database

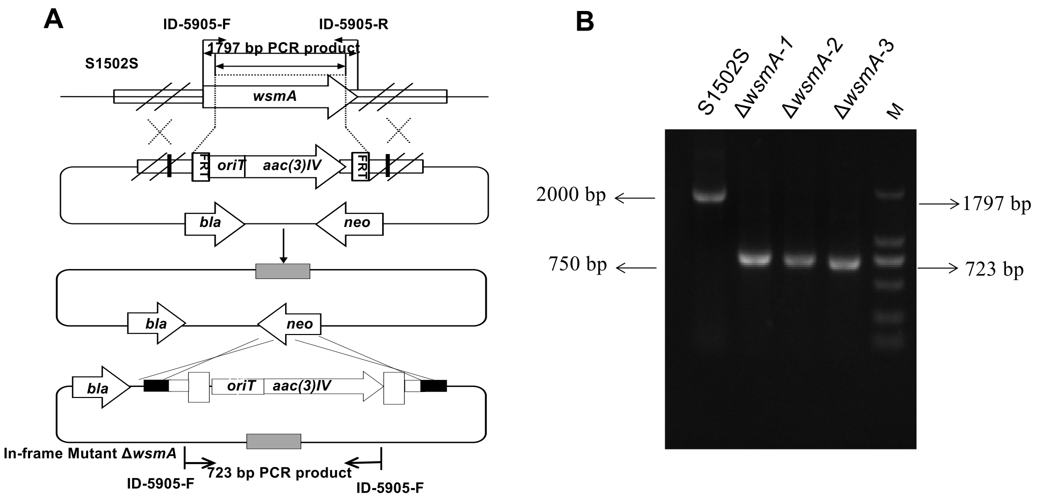

Figure S22. Disruption of *wsmA* in *S.* sp. S1502/Δ*stp1* via PCR-targeting. (A) Schematic representation for disruption of *wsmA*. (B) PCR analyses of the *S.* sp. S1502/Δ*stp1* strain and the *wsmA* double-cross mutant carried out using the primers listed in Table S2. M: DNA molecular ladder; WT: using the genomic DNA of *S.* sp. S1502/Δ*stp1* as template; Δ*wsmA*-1-3: using the genomic DNA of *stp1* mutant as template.

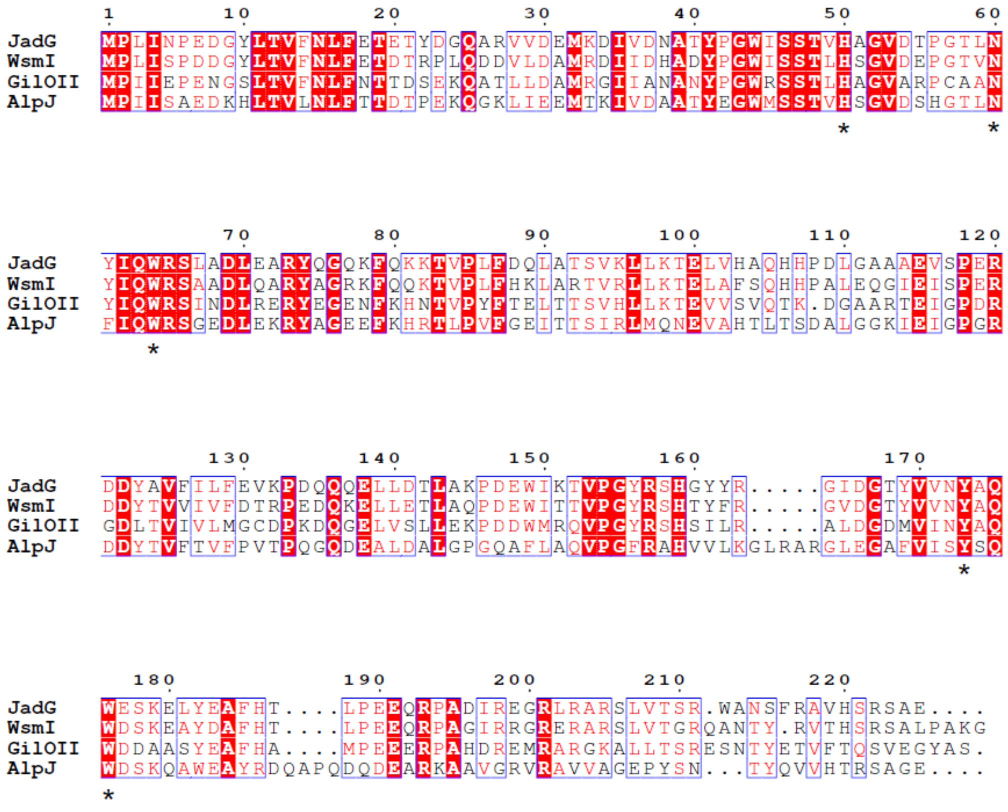

Figure S23 Amino acid sequence alignment of JadG, **WsmI**, GilOII and AlpJ.

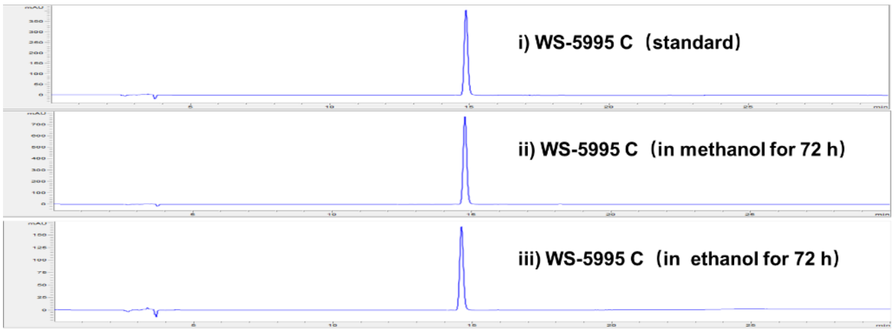

Figure S24 transformation of WS-5995 C.

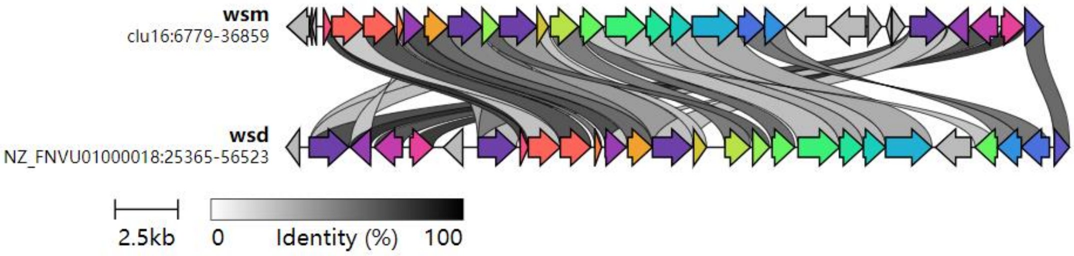

Figure S25 Comparison of *wsm* and *wsd* gene clusters using Clinker.

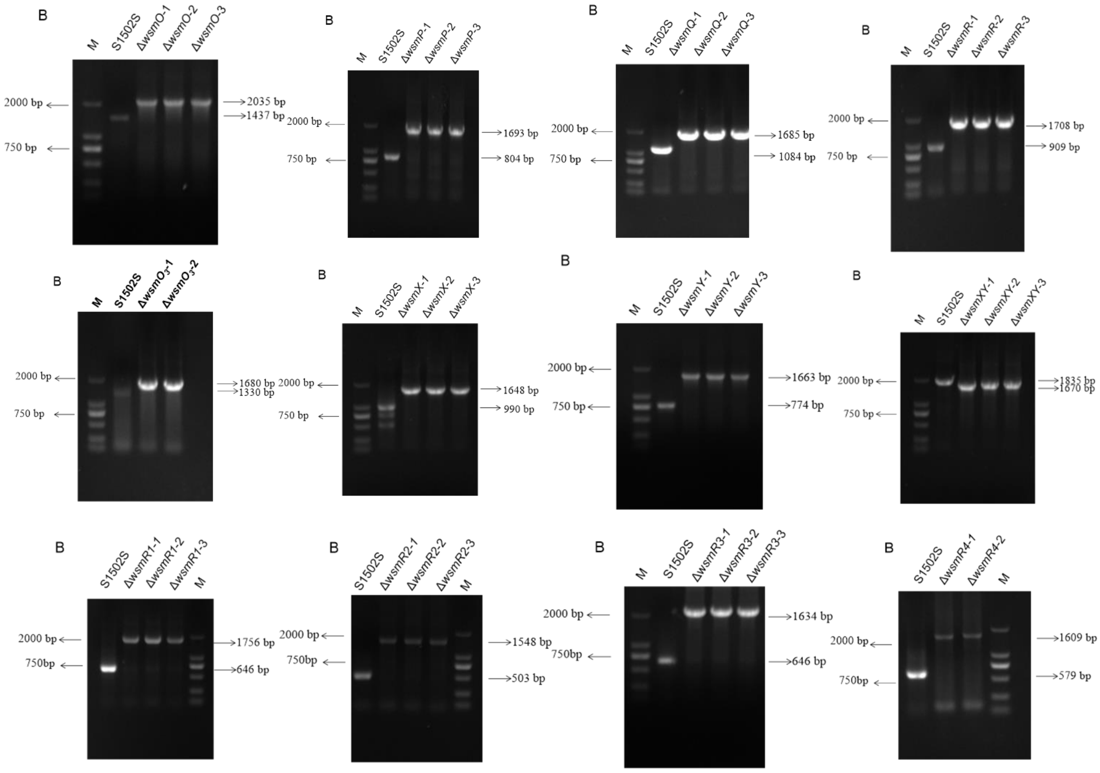

Figure S26 PCR verification of disruption of *genes of wsm* in *S.* sp. S1502/Δ*stp1* via PCR-targeting.

Reference:

1. Wang, Y.; He, J.; Alam, M. S.; Wang, F.; Shang, Z.; Chen, Y.; Sun, C.; Lu, Z.; Gao, Y.; Zhang, T.; Ju, J.; Ma, J., Efficient mutasynthesis of "non-natural" antitubercular ilamycins with low cytotoxicity. *ACS Synthetic Biology* **2024,** 13, (3), 930-941.
2. Luo, M.; Wang, Y.H.; Frisch, D.; Joobeur, T.; Wing, R.A.; Dean, R.A. Melon bacterial artificial chromosome (BAC) library construction using improved methods and identification of clones linked to the locus conferring resistance to melon Fusarium wilt (Fom-2). *Genome* **2001**, *44*, 154–162.
